# Identification of Ohnolog Genes Originating from Whole Genome Duplication in Early Vertebrates, Based on Synteny Comparison across Multiple Genomes

**DOI:** 10.1371/journal.pcbi.1004394

**Published:** 2015-07-16

**Authors:** Param Priya Singh, Jatin Arora, Hervé Isambert

**Affiliations:** CNRS UMR168, UPMC, Institut Curie, Research Center, Paris, France; Ouzounis, Hellas, GREECE

## Abstract

Whole genome duplications (WGD) have now been firmly established in all major eukaryotic kingdoms. In particular, all vertebrates descend from two rounds of WGDs, that occurred in their jawless ancestor some 500 MY ago. Paralogs retained from WGD, also coined ‘ohnologs’ after Susumu Ohno, have been shown to be typically associated with development, signaling and gene regulation. Ohnologs, which amount to about 20 to 35% of genes in the human genome, have also been shown to be prone to dominant deleterious mutations and frequently implicated in cancer and genetic diseases. Hence, identifying ohnologs is central to better understand the evolution of vertebrates and their susceptibility to genetic diseases. Early computational analyses to identify vertebrate ohnologs relied on content-based synteny comparisons between the human genome and a single invertebrate outgroup genome or within the human genome itself. These approaches are thus limited by lineage specific rearrangements in individual genomes. We report, in this study, the identification of vertebrate ohnologs based on the quantitative assessment and integration of synteny conservation between six amniote vertebrates and six invertebrate outgroups. Such a synteny comparison across multiple genomes is shown to enhance the statistical power of ohnolog identification in vertebrates compared to earlier approaches, by overcoming lineage specific genome rearrangements. Ohnolog gene families can be browsed and downloaded for three statistical confidence levels or recompiled for specific, user-defined, significance criteria at http://ohnologs.curie.fr/. In the light of the importance of WGD on the genetic makeup of vertebrates, our analysis provides a useful resource for researchers interested in gaining further insights on vertebrate evolution and genetic diseases.

## Author Summary

Duplication of existing genes with subsequent divergence of duplicated copies has long been recognized as the primary source of genomic innovation. Gene duplication is thus at the root of the evolution and complexification of living organisms. However, gene duplicates have been retained differently depending on the genomic scale of their duplication and their implication in genetic diseases. The scale of genomic duplication spans from small scale segmental duplication to whole genome duplication (WGD), which corresponds to a dramatic doubling event of a species genome. In particular, all vertebrates, including human, descend from two rounds of WGDs, which occurred in their jawless ancestor some 500 MY ago. Interestingly, WGD gene duplicates, also called ‘ohnologs’, have be shown to be more frequently implicated in genetic diseases in human. Hence, identifying ohnologs appears central to better understand the evolution of vertebrates and their susceptibility to genetic diseases. In this study, we present a computational approach to predict ohnologs in six vertebrate genomes, including human, based on the comparison of their local gene content (*i.e.* synteny) with the genomes of six invertebrate outgroups. We show that such synteny comparisons across multiple genomes enhance the statistical power of ohnolog identification compared to earlier approaches.

## Introduction

Gene duplication and their subsequent divergence is the primary source of new genes in eukaryotes. The importance of evolution by gene duplication is exemplified by a large number of paralogous genes in most eukaryotic genomes. In addition to duplication of single genes or genomic segments, duplications of the entire genome have now been firmly established in all major eukaryotic kingdoms. Multiple lineages including unicellular yeast and paramecium, as well as many plants and animals are known to descend from polyploid ancestors, often through multiple rounds of genome duplications [1]. In vertebrates, whole genome duplications (WGD) were first hypothesized by Susumu Ohno [2] (the 2R-hypothesis), after whom WGD duplicated genes are now referred to as *“ohnologs”*.

Interestingly, duplicated genes originating from whole genome duplication have been preferentially retained in different functional categories as compared to duplicated genes originating from small scale duplication [3–6]. In particular, many ohnologs have been retained in gene families involved in development, signaling and gene regulation [3, 7–10], and led to the emergence of novel cell types in vertebrates, such as the neural crest, the midbrain/hindbrain organizer and neurogenic placodes [11]. In addition, ohnologs are frequently associated with diseases such as cancer [3, 5, 6, 12–14], and are particularly prone to dominant deleterious mutations [5, 6] as rationalized from a population genetics perspective [5, 15]. These observations suggest that the identification of ohnologs with high statistical confidence has important implications to better understand the developmental complexity of vertebrates as well as their enhanced susceptibility to dominant deleterious mutations and associated diseases.

**Fig 1:**
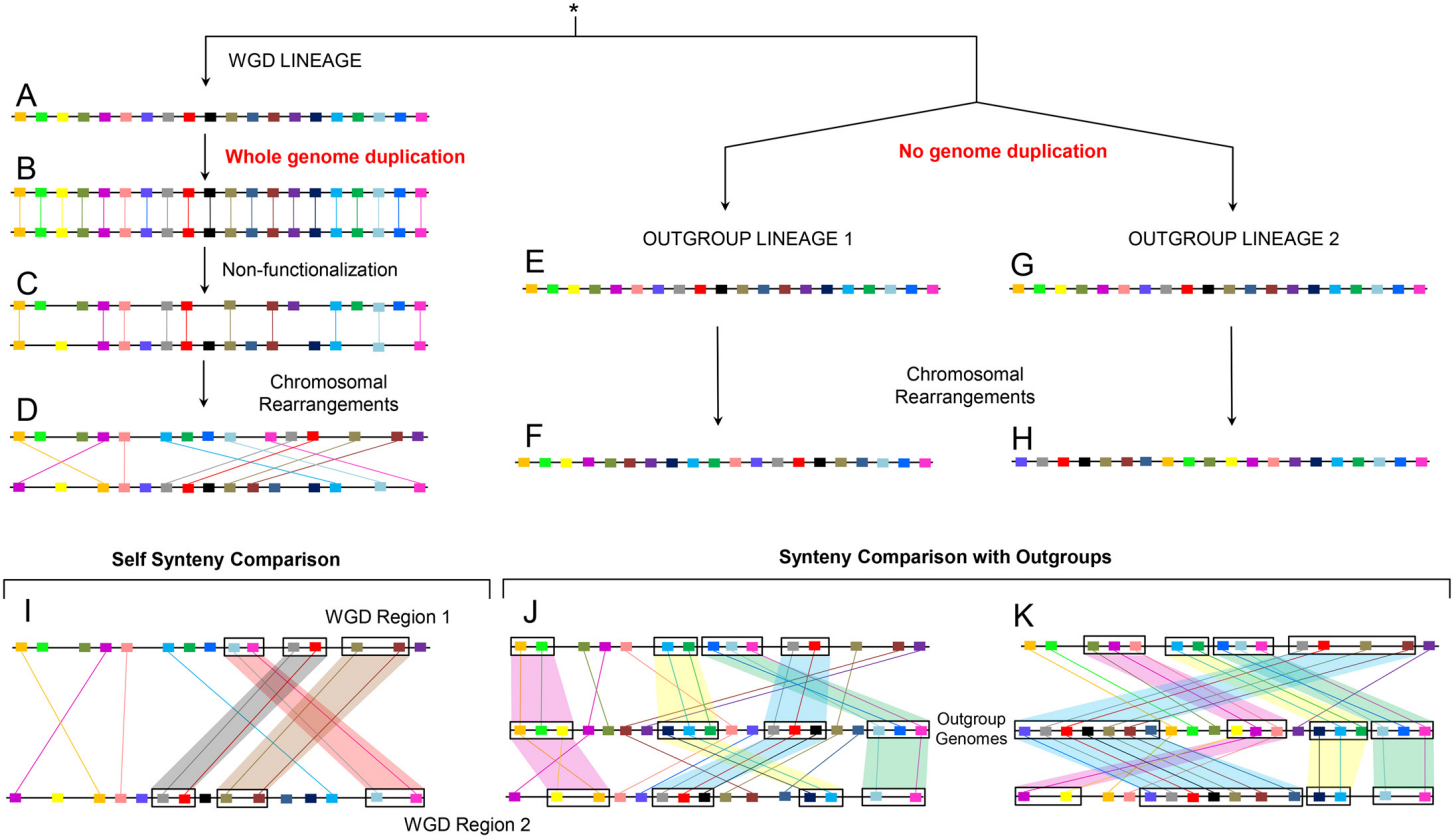
Evolution after WGD and identification of ohnologs. Evolution after WGD and identification of ohnologs using content-based synteny comparison. The genomes of three lineages sharing a common ancestor are shown. Orthologs and paralogs have been depicted by the same color. The WGD lineage (A) underwent whole genome duplication (B) followed by non-functionalization (C) and genome rearrangements (D) leading to the current intragenomic content-based synteny (I). By contrast, the two outgroup genomes without WGD (E, G) experienced lineage specific genome rearrangements (F, H) leading to 1-to-2 content-based synteny pattern with the WGD lineage (J, K). Note, that some ohnolog pairs (D) are only identified by one of the two outgroups (J or K) due to lineage specific rearrangements.

However, the identification of ohnologs in vertebrate genomes is not straightforward [16]. During the millions of years of evolution following WGD, sister regions created by WGD are redistributed across the paleopolyploid genome by chromosomal rearrangements and degenerate by the loss of the majority of ohnologs (Fig 1). In principle, these degenerated WGD duplicated regions sharing a few ohnolog pairs can be identified in the paleopolyploid genome by comparing its genome-wide synteny either with itself (Fig 1I) or with outgroup genomes diverged before the WGD event (Fig 1J and 1K). Yet, the two rounds of WGD at the onset of vertebrates are among the oldest known genome duplications and the conservation of gene order (or micro-synteny) between extant vertebrate and invertebrate outgroup genomes is limited [17]. This makes WGD detection methods based on micro-synteny conservation [18–23] difficult to apply to WGD from early vertebrates. Other methods, not-based on synteny, such as Ks-based methods [24, 25] and more recent phylogenetic methods [26, 27], cannot be easily applied to the 500 MY-old WGD in vertebrates either, due to the saturation effect of the synonymous mutation rates Ks [28] and the difficulty in distinguishing between the two rounds of WGD in the phylogeny of early vertebrates [17, 29].

As an alternative, a number of studies have proposed to identify ohnologs in the human genome by relaxing strict gene-order criteria and searching, instead, for content-based synteny [30] between the human genome and a single invertebrate outgroup genome [17, 31] or within the human genome itself [3, 4, 32]. Using content-based synteny criteria, however, increases the odds of old duplicates being incorrectly identified as ohnologs, if no quantitative assessment of the statistical confidence of ohnolog pair candidates is performed. In addition, performing synteny comparison with a single outgroup may lead to omission of many ‘true’ ohnolog pairs, whose orthologs have moved to different non-syntenic regions in the extant outgroup genome (Fig 1).

In this study, we have extended these latter approaches to six amniote vertebrates (human, mouse, rat, pig, dog and chicken) by investigating the conservation of content-based gene synteny relative to six invertebrate outgroup genomes (lancelet, two seasquirts, sea urchin, fly and worm, S1 Fig). We also analyzed the synteny conservation from the regions created by 2R-WGD within each of the vertebrates, and then integrated the synteny information from both self and outgroup comparisons. The integration of synteny information across multiple genomes enables to identify ohnologs that are no longer in significant synteny in a particular vertebrate genome, as long as their ortholog status can be unequivocally established with proper ohnologs in other vertebrates. We present below the general principles of our multiple genome comparison approach to identify 2R ohnologs and provide a quantitative assessment of the statistical confidence of each ohnolog pairs by comparison with the expected spurious synteny obtained with shuffled genomes. We show that the synteny comparison across multiple genomes enhances the statistical power of ohnolog identification in vertebrates compared to earlier approaches. The resulting ohnolog pairs and families are accessible at http://ohnologs.curie.fr/ for three statistical confidence levels and can also be recompiled for specific, user-defined, significance criteria.

## Methods

### Overview of the approach

We implemented content-based synteny comparisons between each amniote vertebrate and multiple invertebrate outgroup genomes. Initial ohnolog candidates were identified, in each vertebrate genome, using a window-based approach to detect putative synteny blocks between each vertebrate and the six outgroup genomes (outgroup comparison, Fig 1J), extending earlier similar approaches [17, 30, 31]. Additional synteny block candidates were also identified by comparing each vertebrate genome to itself (self comparison, Fig 1I) [3, 32] and ohnolog pair candidates were further restricted to paralogous pairs duplicated at the base of vertebrates according to Ensembl compara [33–35] (see S1 Text, Supplementary Materials and Methods). S1 Fig lists the numbers of human ohnolog pair candidates identified by each invertebrate outgroup and human-human synteny comparison, before applying any filtering on the statistical support of candidate synteny blocks. We identified a total of 15,107 such putative ohnolog pair candidates, including 11,428 identified with at least one outgroup and 15,054 identified by self comparison alone.

**Fig 2:**
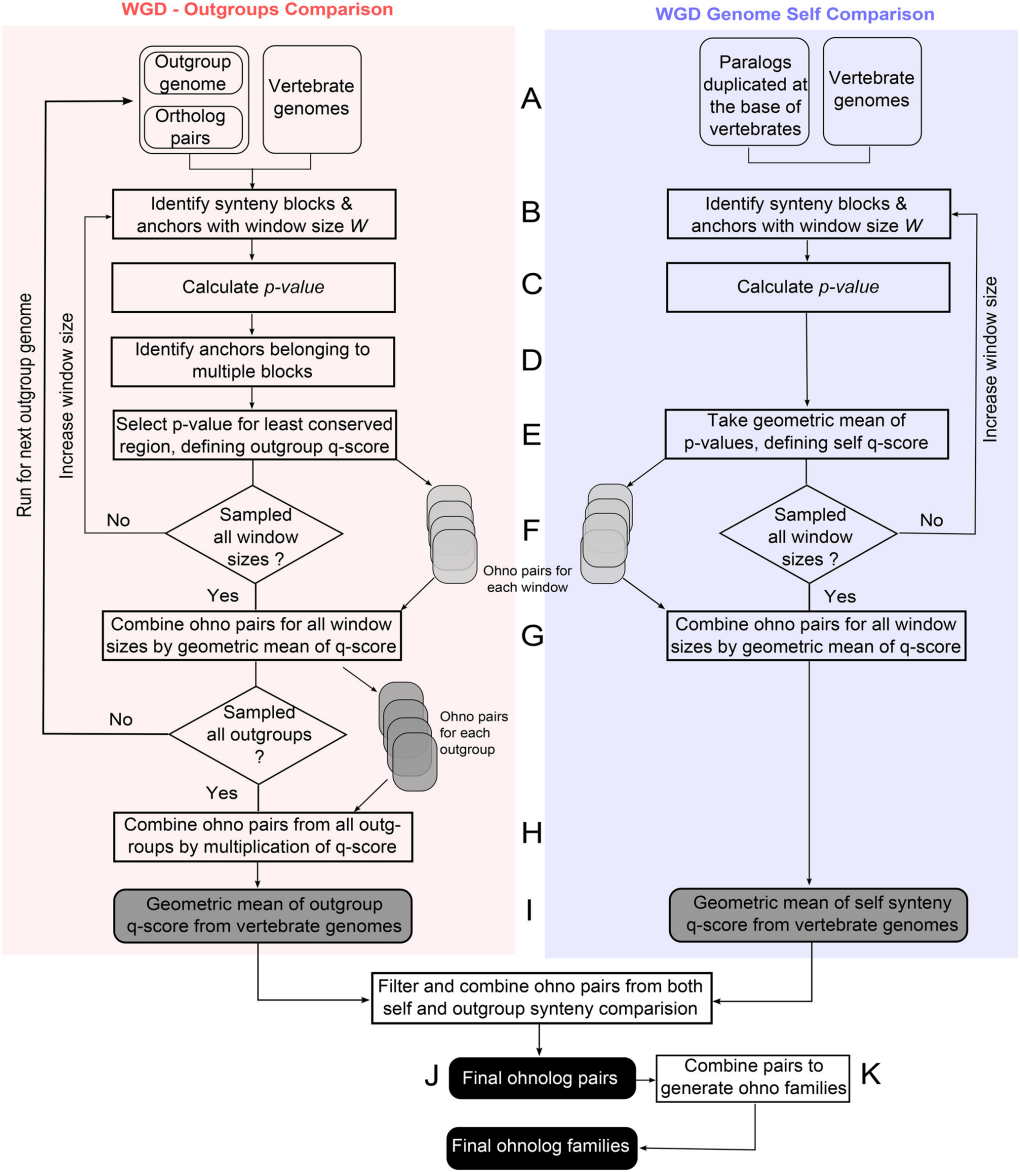
Flowchart of the algorithm to identify ohnologs. Flowchart of the algorithm to identify ohnolog pairs and construct ohnolog families for a single vertebrate genome using content-based synteny comparison with multiple outgroup genomes (left panel) and self-comparison (right panel), see main text and S1 Text for details.

To narrow down this initial list of ohnolog candidates, we developed a quantitative approach to assess the statistical confidence of each ohnolog pair candidate. This quantitative approach and corresponding ‘q-score’, ranging from 0 to 1, estimates the probability that each ohnolog pair is simply identified by chance. Hence, lower q-scores imply more statistically significant ohnolog pairs (see S1 Text). Finally, we integrated q-scores for outgroup-comparison and self-comparison from all vertebrates, and filtered the ohnolog pairs based on the resulting combined q-scores. A flowchart summarizing our algorithmic approach is depicted in Fig 2. The pipeline of the approach is outlined below with methodological details described in Supplementary Materials and Methods (S1 Text).

### Outline of the computational pipeline

1. **Initial ohnolog candidates from comparison with six outgroup genomes.** Initial ohnolog candidates in each amniote genome were identified using a window-based approach to detect putative synteny blocks between each vertebrate genome and the six outgroup genomes (S4 Fig). We used the orthologs between each vertebrate and outgroup genomes to identify conserved synteny blocks for a given window size *W* ranging from 100 to 500 genes (Fig 2A and 2B, left panel). Vertebrate genes that lie on such synteny blocks and share the same outgroup ortholog (1-to-2 synteny conservation pattern) are ohnolog candidates from the outgroup comparison (S5A Fig, Fig 2D).
2. **Initial ohnolog candidates from self-comparison in each amniote genome.** Additional ohnolog candidates were also identified through self-comparison in each amniote genome using the same window size *W* (Fig 2A and 2B, right panel). We identified regions in each vertebrate genome with multiple paralogs duplicated at the base of vertebrates (S5B Fig).
3. **Filtering ohnolog candidate pairs by duplication time.** Ohnolog pair candidates from both outgroup and self-comparison are further restricted to paralogous gene pairs duplicated at the base of vertebrates according to Ensembl compara (see S1 Text).
4. **Calculating P-value and q-score for synteny blocks.** A P-value for each synteny block candidate for outgroup and self comparisons is derived based on the observed number of homologous gene pairs in the defined window. This P-value assesses the chance that the observed numbers of orthologous or paralogous gene pairs are unlikely to result simply by chance, due to the average and variance of gene pairs across synteny windows (S6 Fig, Fig 2C). We then combine P-values to define quantitative scores or ‘q-scores’ for outgroup and self comparisons to assess the statistical significance of each ohnolog pair (S1 Text, Fig 2E).
5. **Averaging across different window sizes.** The ohnolog identification and statistical significance analysis are subsequently performed for five different window sizes ranging from 100 to 500 genes and a global q-score for outgroup and self comparison is obtained through geometric average for each ohnolog pair over the different window sizes (Fig 2F and 2G).
6. **Leveraging statistical power of multiple outgroup comparison.** To take advantage of the statistical power of multiple outgroup comparison, q-scores computed from the different outgroup comparisons are simply multiplied to lead to a unique, more significant global q-score taking into account all outgroups. This amounts to assume independent rearrangements in each outgroup lineages, which diverged more than 500 MY ago. Comparisons with randomized genomes confirmed limited spurious identification of false positive ohnologs due to outgroup genome correlations (S1 Text, S7 Fig and Fig 2H).
7. **Computing consensus amniote ohnologs.** The statistical power of multiple genome comparison is further exploited to obtain a consensus set of amniote ohnologs. To this end, outgroup and self-synteny q-scores of ohnolog pairs from different amniotes are averaged over all genomes with corresponding ortholog pairs in Ensembl, S1 Text. Using averaged q-scores enables to circumvent some recent lineage specific rearrangements in amniote genomes, while taking into account their long common evolutionary history since divergence from invertebrate outgroups (Fig 2I).
8. **Defining statistical confidence criteria.** We then construct three sets of ohnologs by combining averaged q-scores from both outgroup (${\bar{Q}}_{outgr}$) and self (${\bar{Q}}_{self}$) comparisons to define three significance criteria (Fig 2J),
  - **Strict**:    ${\bar{Q}}_{outgr}<0.01$ AND ${\bar{Q}}_{self}<0.01$
  - **Intermediate**: ${\bar{Q}}_{outgr}<0.05$ AND ${\bar{Q}}_{self}<0.3$
  - **Relaxed**:   ${\bar{Q}}_{outgr}<0.05$ OR (${\bar{Q}}_{outgr}\;<\;0.5$ AND ${\bar{Q}}_{self}\;<\;0.01$)
  Note that the relaxed criteria may also include a number of paralogs from large scale segmental duplications from the origin of vertebrates.
9. **Generating ohnolog gene families.** Finally, we construct ohnolog gene families using a depth-first search algorithm [36] in the space of ohnolog pairs (S1 Text, Fig 2K).

## Results/Discussion

### Human ohnologs

The strict, intermediate and relaxed criteria lead to three sets of ohnolog pairs in the human genome with decreasing statistical confidence levels: 2,695 ohnolog pairs with very high confidence, 4,827 with high confidence and 8,178 with medium confidence, respectively (Table 1). These predicted ohnolog pairs are also significantly different from ohnolog pairs reported in earlier studies [3, 4], Table 1. In particular, 617 (23%) of the 2,695 strict ohnologs pairs from our analysis are not identified in [3]. For example, the strict ohnolog pairs between the transcription factors *SOX11* and *SOX12* or between the microtubule-associated proteins *MAP2*, *MAP4* and *MAPT* are missing in [3]. Conversely, 3,695 (44%) of the 8,383 ohnolog pairs reported in [3] are excluded by the present analysis. More precisely, we found that 1,853 (50%) of these 3,695 ohnolog pairs ruled out by our analysis have not been duplicated at the base of vertebrates according to Ensembl compara, while 813 (22%) discarded ohnolog pairs are not supported by our quantitative multi-genome synteny comparison and the remaining 1,029 (28%) are excluded by both duplication timing and quantitative multi-genome synteny assessment. For example, the 3-oxoacid CoA-transferase genes *OXCT1* and *OXCT2*, previously reported as ohnologs [3], have in fact been duplicated more recently than the 2R-WGD (*i.e.* in mammals according to Ensembl compara). By contrast, the signaling genes *WNT1* and *WNT3*, also reported as an ohnolog pair [3] are not supported by our quantitative multi-genome synteny criteria and have also been duplicated earlier than the 2R-WGD (*i.e.* in bilateria or coelomata according to Ensembl compara).

**Table 1:** Individual ohnologs, pairs and families for different quantitative criteria in the human genome (see text).

Confidence criteria (this study) *vs* earlier studies	Ohno Pairs	Individual Ohnologs	Ohnolog Families	Family Sizes				% of families with size ≤ 4
				2	3	4	≥ 5	
Strict criteria	2695	3544	1381	970	321	83	7	99.5%
Intermediate criteria	4827	5504	2024	1337	481	175	31	98.5%
Relaxed criteria	8178	7831	2642	1676	633	245	88	96.7%
Makino & McLysaght 2010	8383	6993	2351	1475	547	214	115	95.1%
Huminiecki & Heldin 2010	29344	9557	2543	1222	618	332	371	85.4%

**Fig 3:**
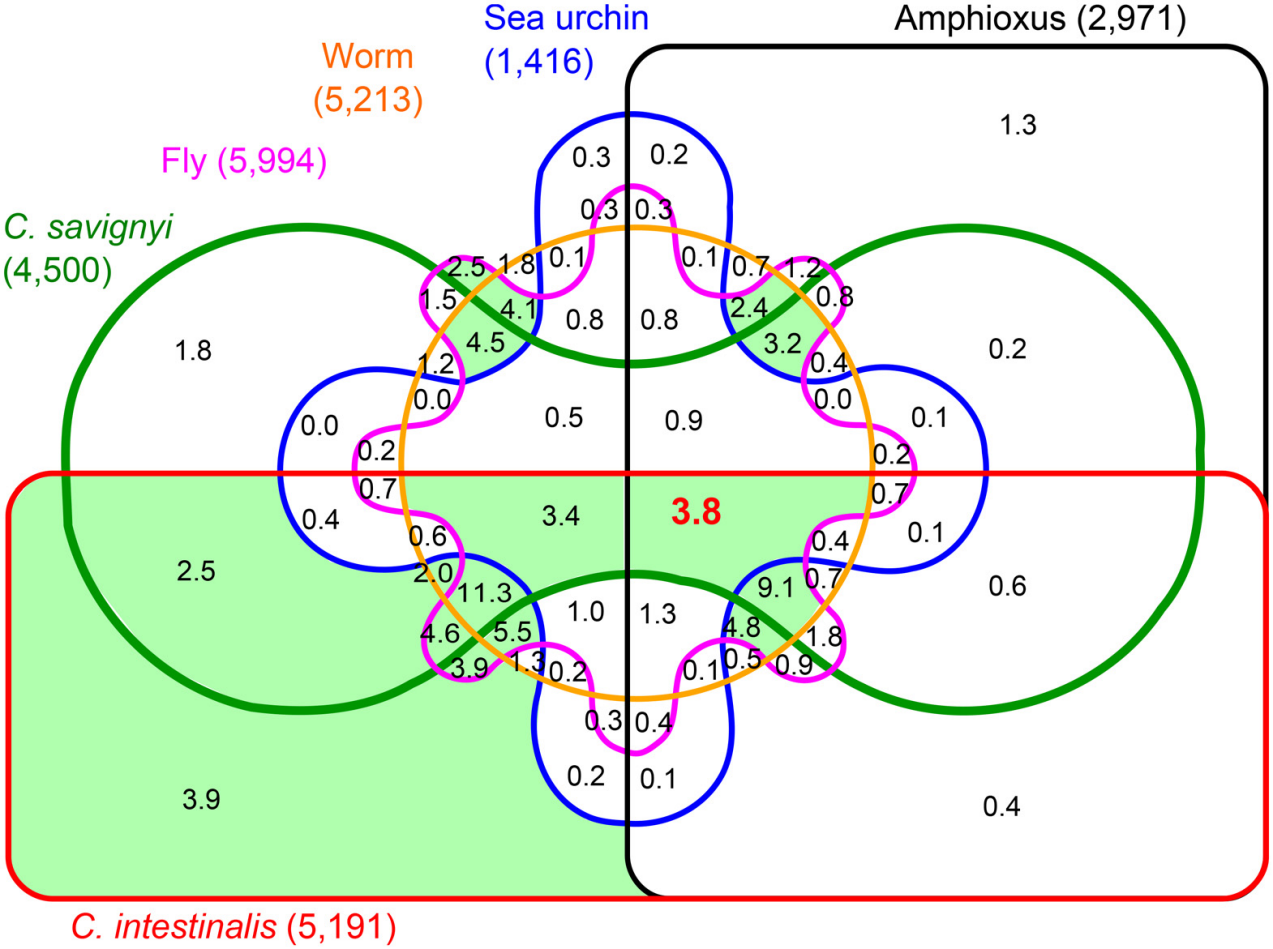
Venn diagram of distribution of human ohnologs with respect to outgroups. A six-way Venn diagram showing the distribution in percentages of the 7,715 of the total 8,178 human ohnolog pairs that are identified by at least one outgroup and predicted from the relaxed criteria. Only 3.8% of human ohnolog pairs are identified by all outgroup. Each of the shaded sectors in green contributes to more than 2% of all ohnolog pairs (numbers of ohnolog pairs are given in S8 Fig).

The distribution of our ohnolog pairs with respect to all six outgroups is depicted on a six way Venn diagram in Fig 3 (percentages) and S8 Fig (numbers). Ohnolog pairs range from 1,416 with sea urchin comparison to a maximum of 5,994 using *Drosophila melanogaster* as outgroup. There are only 3.8% (293) ohnolog pairs identified by all outgroups, while each outgroup combination shaded in green in Fig 3 contributes to more than 2% of the total number of ohnolog pairs. This illustrates that many ohnologs would not be identified using just a single outgroup genome owing to lineage specific rearrangements in the outgroup genomes, limitations of genome assembly/annotation or homology criteria. In particular, while 90% (6,943) ohnolog pairs in human are identified by at least one chordate outgroup genome, 10% (772) ohnolog pairs are only identified by synteny comparison with non-chordate genomes. For example, the homeobox protein ohnolog pair *VAX1*/*VAX2* and the nuclear receptor co-repressor ohnolog pair *LCOR*/*LCORL* are only identified by synteny comparison with *D. melanogaster* and *C. elegans*.

The final human ohnolog counts for strict, intermediate and relaxed criteria are respectively, 3,544 ohnologs (Strict Criteria); 5,504 ohnologs (Intermediate Criteria) and 7,831 ohnologs (Relaxed Criteria), Table 1. This is also to be contrasted with the results of previous studies that used either content-based synteny comparison with a single outgroup [17, 31] or only self comparison [3, 4, 32] without statistical significance criteria to filter out spurious synteny block conservation. We found that the available sets of human ohnologs from these early studies also present significant differences from our results. For instance, the set of 7,075 ohnolog genes from [3] shows significant differences from ours (S9 Fig), as 14%, 18% and 23% of our human ohnologs for strict, intermediate and relaxed criteria, respectively, have not been identified in [3]. Conversely, 57%, 33% and 15% of this early ohnolog data set are excluded from our strict, intermediate and relaxed human ohnolog sets, respectively (S9 Fig). As discussed above, this is due to inconsistent duplication times, according to Ensembl Compara, and/or limited statistical supports for each confidence criteria.

We then reconstructed ohnolog families from ohnolog pairs using a depth first search algorithm [36] (S1 Text). The resulting ohnolog families also contain paralogs which are small scale duplicates with respect to each other but form ohnolog pairs with a third gene of the family. Accounting for such small scale duplicates, eventually lead to ohnolog families with an expected maximum of four ohnologs retained from the two rounds of WGD in early vertebrates. However, as most genes lose their duplicates after WGD, most ohnolog families are expected to be of size two or three.

We obtained 1,381, 2,024 and 2,642 ohnolog families using strict, intermediate and relaxed criteria, respectively, for the human genome. Most remarkably, for almost all of these families, the size never exceeds four ohnologs, as expected for two rounds of WGD. As depicted in Table 1, all but 7 ohnolog families (99.5%) have a size smaller or equal to four for the strict criteria. Even with the most relaxed criteria, 96.7% of ohnolog families are consistent with a maximum family size of four ohnologs. Furthermore, a sharp decline in the number of families was observed beyond size four, suggesting a limited number of false positive ohnologs incompatible with two rounds of genome duplications. Interestingly, however, many three- or four-ohnolog families could not be identified independently in individual amniote genomes, but only by integrating synteny information from different amniote genomes, such as the four-ohnolog family *ERAS*/*HRAS*/*KRAS*/*NRAS* (relaxed criteria).

We also applied the same approach to generate ohnolog families from the ohnolog pairs provided by [3] and [4]. 95.1% of ohnolog families from [3] are consistent with two rounds of WGD and only 85.4% of ohnolog families from [4] have sizes up to four ohnologs. Clearly families exceeding four ohnologs must result either from the erroneous concatenation of distinct ohnolog families or include non-ohnolog genes. For instance, the ohnolog status of *TRPV5* and *TRPV6* [3] from the large family of six ion channels (*TRPV1-6*) are not supported by our quantitative assessment of self- and outgroup synteny. Conversely, we could also identified previously overlooked ohnologs, through high confidence assessment of self- and outgroup synteny. For instance, the guanine exchange factor *RGL2* was found to be part of a four-ohnolog family with strict criteria, *RGL1*/*RGL2*/*RGL3*/*RALGDS, RGL4* (with *RGL4* a small scale duplicate of *RALGDS*).

### Ohnologs in other amniote vertebrates

In addition to the human genome, our synteny comparison approach across multiple genomes also identified ohnologs in five other amniote genomes: four mammals (mouse, rat, pig and dog) and one bird (chicken). Starting from ohnolog pairs in each species, the same approach was used to generate ohnolog families. A summary of individual ohnologs, ohnolog pairs and ohnolog families for these genomes is given in S2 Fig for strict, intermediate and relaxed quantitative criteria.

**Fig 4:**
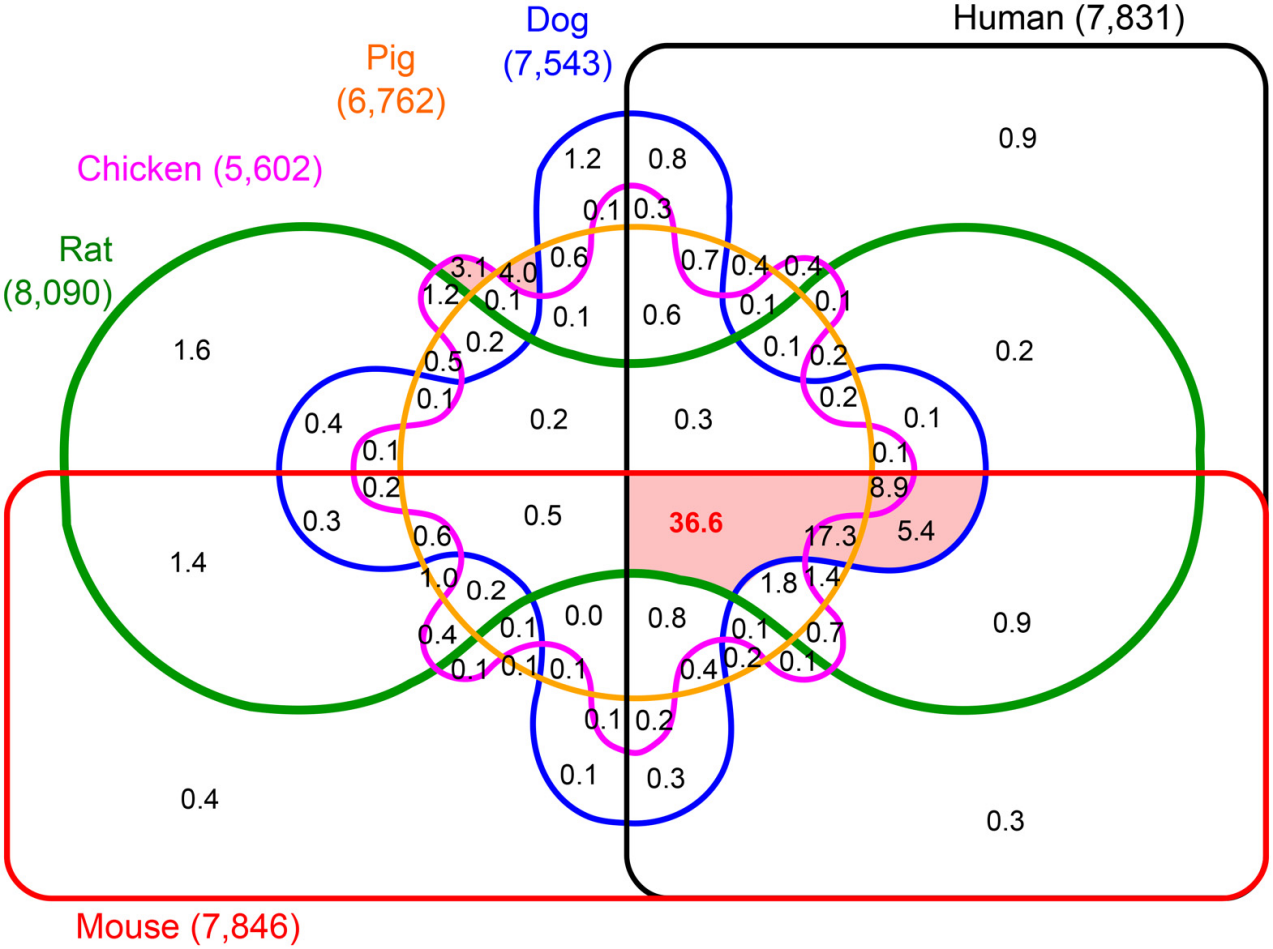
Venn diagram of the distribution of amniote ohnologs. A six-way Venn diagram showing the distribution in percentages of the ohnologs identified in at least one amniote and predicted from the relaxed criteria. 36.6% of ohnologs are found in all six amniotes. Each shaded sectors in red contributes to more than 2% of all consensus ohnologs in amniotes (numbers of ohnologs are given in S10 Fig).

The level of annotation of these genomes is variable and the number of annotated protein coding genes range from 15,310 for chicken to 22,865 for the rat genome (S3 Fig). Using the relaxed criteria, a minimum of 4,282 to a maximum of 9,708 ohnolog pairs could be identified for chicken and rat, respectively. The six way Venn diagram in Fig 4 summarizes the fractions of retention *versus* lineage specific loss of ohnologs in the analyzed amniote genomes for the relaxed criteria (see S10 Fig for ohnolog numbers). Statistics for the strict criteria are given in S11 Fig. The identification of consensus ohnologs in this context implies that we are able to detect their ohnolog status through self- and outgroup synteny comparison or, alternatively, through orthology with *bona fide* ohnologs in other amniotes (see S1 Text). Indeed, ohnologs that are no longer in significant synteny in a particular vertebrate genome can still be identified, as long as their ortholog status can be unequivocally established with proper ohnologs in other vertebrates. This enables to circumvent strict synteny conditions in a specific genome.

By contrast to the small fraction of ohnolog genes identified by the six outgroups (*i.e.* 3.8%, Fig 4), 36.6% of predicted ohnologs are shared by all six amniotes, 53.9% by the five mammals and 74.3% by human, mouse and rat, while only a few other combinations of specific amniotes contribute to more than 2% of all ohnologs (see sectors shaded in red in Fig 4). This illustrates that the ohnologs have been largely conserved in mammals and to a lesser extent across amniotes. Likewise, ohnolog family sizes in each amniote genome consistently follow similar distributions as observed in human (Table 1) with a sharp decline in the number of families beyond the maximum size of four ohnologs (S2 Fig). In fact, the numbers of ohnologs in each family are most often the same in human and other mammals (in particular mouse) with occasional differences, typically missing ohnologs, in chicken which has significantly fewer genes (including ohnologs) than other amniotes considered in this study. For example, chicken has lost a number of adipokine genes [37] such as *SERPINE1*, which is part of a four-ohnolog family in mammals, *SERPINE1*/*SERPINE2*/*SERPINE3*/*SERPINI1*|*SERPINI2* (where *SERPINI1* and *SERPINI2* are small scale duplicates). Similarly, all three ohnolog genes in the family of DNA binding Forkhead box protein A, *i.e.* *FOXA1*/*FOXA2*/*FOXA3*, are missing in the annotated chicken genome. Hence, differences in the shared ohnologs in Fig 4 arise due to lineage specific ohnolog loss or, possibly, due to missing annotations of genes and/or orthologs in these genomes.

We have so far restricted our synteny conservation analysis across multiple genomes to selected amniote genomes. In particular, amphibians and fishes have not been included in the analysis. This is because assembled chromosomal scaffolds of available amphibians (*e.g.* Xenopus) and non-teleost fishes (*e.g.* elephant shark and coelacanth) do not contain enough genes to be included in a content-based synteny conservation analysis (*e.g.* 81% of *X. tropicalis* genes are on chromosomal scaffolds with fewer than 50 genes). As for teleost fish genomes, they experienced a third more recent (3R) WGD, about 300 MY ago [38] in addition to the two rounds of (2R) WGD common to all vertebrates. This additional 3R WGD implies methodological issues specific to teleost fish genomes, which will be addressed in a forthcoming extension of our computational approach to identify ohnologs through multiple genome synteny comparison.

### Ohnologs association with functional categories and diseases

**Fig 5:**
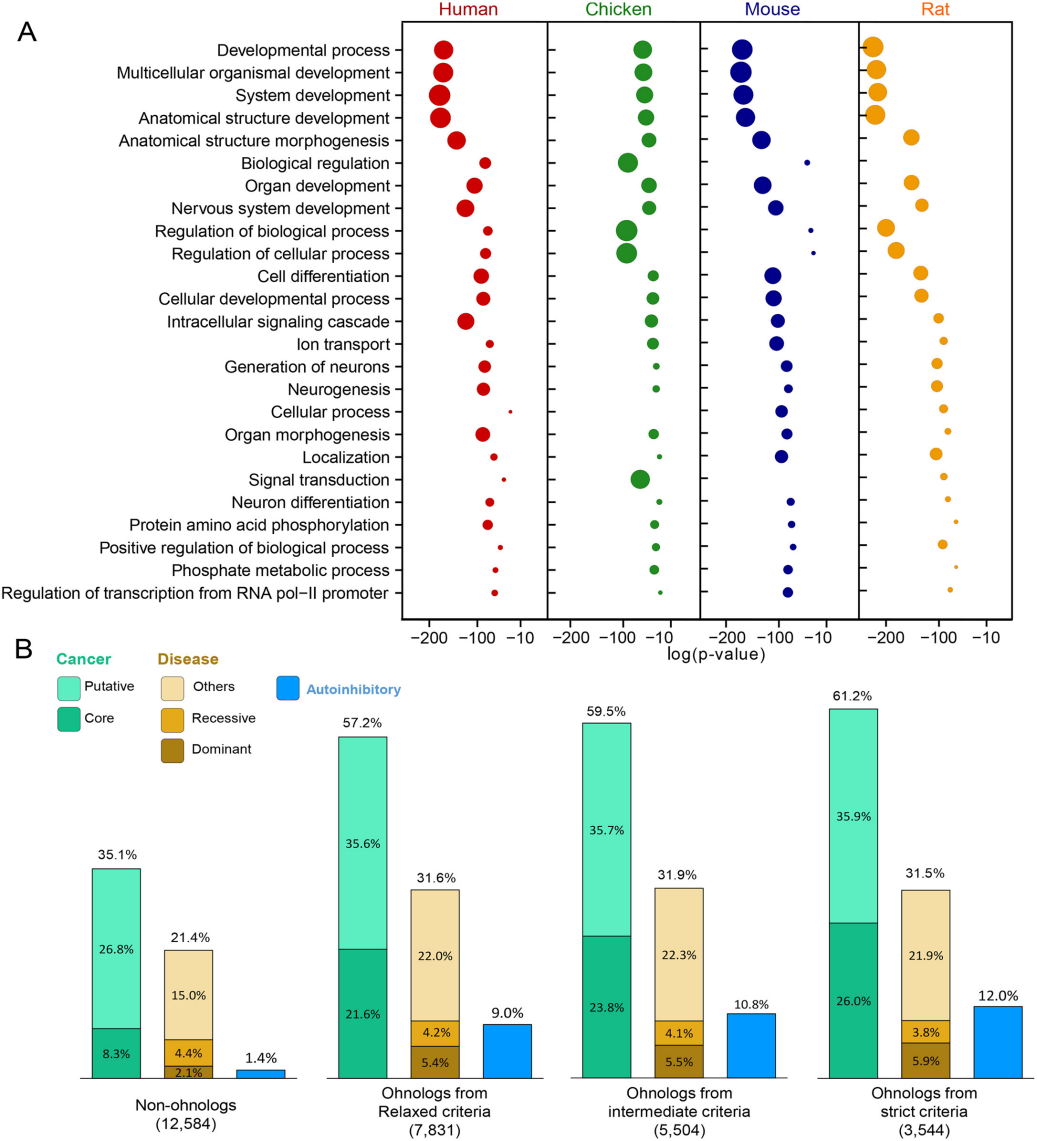
Ohnolog association to cancer and diseases in human. (A) Gene Ontology enrichment for four amniote ohnolog datasets from the relaxed criteria. From top to bottom, the top 25 enriched GO terms, sorted on the basis of average rank across the four genomes. Bubble sizes are proportional to the rank (p-value) of the term for each genome. (B) Ohnolog association to cancer and genetic diseases in human. Ohnolog enrichment is especially significant for core cancer genes, autosomal dominant disease genes and genes with autoinhibitory protein folds, see text, in agreement with earlier reports [5, 6, 15].

As outlined in the introduction, ohnologs have been reported to be preferentially retained in functional categories associated with development, signaling and gene regulation in the human genome [3, 7–10]. We performed a Gene Ontology (GO) enrichment analysis on four amniote vertebrates using DAVID [39] and observed the same general trend across these amniote genomes (Fig 5A). This confirms that ohnologs are associated with similar functional categories in different vertebrates.

In addition, ohnologs have also been associated with disease mutations [5, 12–14], in particular with dominant deleterious mutations frequently implicated in cancers and dominant genetic diseases [5, 6, 15]. Fig 5B confirms such cancer and genetic disease associations for all three ohnolog confidence criteria adopted in this study. This is particularly significant for core cancer genes [5, 40] (amounting for just 8.3% of non-ohnologs but up to 21.6–26% of ohnologs, *i.e.* a 2.6–3.1 fold increase, *p* = 3.4 × 10^−153^ Fisher Exact Test) and autosomal dominant diseases (amounting for just 2.1% of non-ohnologs but up to 5.4–5.9% of ohnologs, *i.e.* a 2.6–2.8 fold increase, *p* = 3.4 × 10^−27^ Fisher Exact Test) in agreement with earlier reports [5, 6] and evolutionary models [15]. We also analyzed the enrichment of ohnologs in genes with autoinhibitory protein folds, which are prone to dominant deleterious mutations. To this end, we collected genes with autoinhibitory protein folds either from careful literature curation [5] or based on the annotation of structural domains frequently associated with autoinhibition (*i.e.* SH3, DH, PH, CH, Drf and Eth domains), identified using Hidden Markov Model (HMM) search [41] against the PFAM database [42] (see Supplementary Methods). We observed that the ohnologs are particularly enriched in genes with autoinhibitory protein folds (amounting for just 1.4% of non-ohnologs but up to 9–12% of ohnologs, *i.e.* a 6.4–8.6 fold increase, *p* = 4.4 × 10^−150^ Fisher Exact Test) [5].

### The ‘Ohnologs’ server

The data of all the ohnolog pairs and families for the six vertebrate genomes is accessible through the ‘Ohnologs’ server at http://ohnologs.curie.fr/. There, users can **i)** search for a particular gene, **ii)** browse pre-compiled ohnolog families and ohnolog pairs or **iii)** generate ohnolog families based on their own, user-defined, quantitative filters. The server is implemented in Perl-CGI and is hosted on a virtual machine at Institut Curie.

On the *Search* page (S12 Fig), the user can search for a gene of interest in any of the six available vertebrates using either Ensembl Id, gene symbol or any desired keywords. Search by functional categories is also possible using Gene Ontology Id or term. If a keyword search does not match any gene directly, we display all the genes matching that keyword in gene symbol, text description or GO term. A hyperlink from this page directs to the details on its ohnolog families and its possible association with human diseases points to Genecards [43] and Cosmic [44] databases. This page also contain links to details in UniProt and Entrez databases if available. If the gene exists in our analysis, and is an ohnolog, users are directed to the details about ohnolog families for each statistical confidence levels (*i.e.*, strict, intermediate and relaxed criteria), S13 Fig.

Alternatively, users can also generate ohnolog families using our multi genome comparison analysis, for any of the six available vertebrate genomes using an arbitrary, user-defined, quantitative criteria for the outgroup and self comparisons. The default values correspond to the strict criteria. The result pages display all the pre-calculated or custom generated families, which can also be downloaded.

In the light of the importance of ohnologs in the evolution of vertebrates and their enhanced association with diseases, our analysis provides a useful resource to gain further insights on the impact of WGD in extant vertebrates.

## Supporting Information

S1 Text Supplementary materials and methods including details on ohnolog identification and analysis. (PDF)

S1 Fig Number of human ohnolog candidates. Number of human ohnologs identified by outgroup and self comparison before applying any quantitative filter for content-based synteny.
(TIF)

S2 Fig Ohnologs in the five non-human amniote genomes analyzed. Individual ohnologs, pairs and families for the three quantitative criteria in the five non-human amniote genomes analyzed.
(TIF)

S3 Fig Numbers of protein coding orthologs and paralogs. Number of protein coding genes, orthologs and paralogs for the analyzed vertebrate (A) and invertebrate (B) genomes.
(TIF)

S4 Fig Schematic tree for the organisms analyzed in this study. Schematic tree for the paleopolyploid and outgroup organisms with duplication nodes taken from Ensembl Compara [33–35]. Gray nodes are not part of Ensembl. Paleopolyploid vertebrate genomes included in this study are highlighted with a red box and invertebrate outgroups (for the 2R-WGD) are highlighted by a green box.
(TIF)

S5 FigIdentification of content-based synteny.Comparison of genomic regions to identify anchor pairs (in red) and ohnolog candidate pairs (dashed red). Each block represents a gene labeled by *O*_*i*_ on the outgroup genome and *V*_*i*_ on the vertebrate genome. Duplicated regions in the vertebrate genome are marked by $V_{1}^{\prime }-V_{n}^{\prime }$. Other orthologous (A) and paralogous (B) relations are depicted by green lines.
**(A)** Identification of synteny *anchors* between an outgroup window and two windows in the vertebrate genome. Using a window of size 8(+1) centered around the *O*_7_−*V*_7_ and $O_{7}-V_{7}^{\prime }$ orthologous pairs, we observe 4 and 3 additional gene pairs between the outgroup and the vertebrate regions 1 and 2, respectively. Hence, *O*_7_−*V*_7_ and $O_{7}-V_{7}^{\prime }$ are two *anchors* sharing the same outgroup ortholog *O*_7_. Hence $V_{7}-V_{7}^{\prime }$ are inferred to be an ohnolog pair candidate, which will be further filtered with quantitative statistical significance criteria or q-score, $Q_{outgr}$, see text.
**(B)** Identification of ohnologs between two regions in the same vertebrate genome. The anchor $V_{7}-V_{7}^{\prime }$ having four additional paralog pairs between the windows, it is directly taken as an ohnolog pair candidate, to be further filtered with quantitative statistical significance criteria or q-score, $Q_{self}$, see text.(TIF)

S6 Fig Principle of P-value calculation between putative synteny blocks. The calculation of *P*_*i*_ for an outgroup gene *O*_*i*_. Illustration of the likelihood calculation, *P*_*i*_, for an outgroup gene *O*_8_ to have an ortholog gene in the vertebrate window (*V*_16_−*V*_20_) defined by the anchor pair (*O*_7_−*V*_18_). *O*_8_ has 5 orthologs in the vertebrate genome: *V*_1_, *V*_8_, *V*_19_, *V*_23_ and *V*_32_. There are 12 possible window locations (highlighted in blue) without any of these orthologs in the vertebrate genome. *P*_*i*_ for this anchor then becomes 1 − 12/31 = 0.6, where 31 is the total number of possible windows on this schematic vertebrate genome (*N*−*W*).
(TIF)

S7 Fig Comparisons of q-score distribution from original and randomized genomes. Comparisons of the global q-score distributions from the original (blue) and randomized (red) genomes; (A) without worm and fly outgroups; (B) with all six outgroup genomes.
(TIF)

S8 Fig Venn diagram of outgroup identification of ohnolog pairs in human. A six-way Venn diagram showing the distribution in numbers of the 7,715 human ohnolog pairs identified by at least one outgroup and predicted from the relaxed criteria.
(TIF)

S9 Fig Comparisons of human ohnologs with Makino-McLysaght dataset [3]. Comparison of our human ohnolog prediction for the three quantitative criteria (strict, intermediate and relaxed, see main text) and the ohnolog dataset from [3].
(TIF)

S10 Fig Venn diagram of distribution of amniote ohnologs for the relaxed criteria. A six-way Venn diagram showing the distribution in numbers of the ohnologs identified in at least one amniote and predicted from the relaxed criteria.
(TIF)

S11 Fig Venn diagram of distribution of amniote ohnologs for the strict criteria. A six-way Venn diagram showing the distribution in numbers (A) and percentages (B) of the ohnologs identified in at least one amniote and predicted from the strict criteria.
(TIF)

S12 Fig Search page on the ‘Ohnologs’ server. (TIF)

S13 Fig Ohnolog family page on the ‘Ohnologs’ server. The result page of the ohnolog family search for the human EMR3 gene is depicted. Families from all three quantitative criteria are displayed, see text. Using the strict criterion, a family of size 2 is generated where *ELTD1 & LPHN2* are ohnologs with *EMR2, EMR3 & LPHN1*. Relaxing the q-score to the intermediate criteria results in an additional ohnolog in this family, *EMTR1*; and to the relaxed criteria results in a family of size 4. Ohnolog partners for the families are displayed in different columns. Genes within the same cell are small scale duplicates *e.g.* *ELTD1—LPHN2*. We use two different separators for SSDs: a comma (,) to distinguish if it is a recent SSD (after 2R-WGD), and a pipe (|) for an ancient SSD (before or around the same time as the 2R-WGD). Hence, *ELTD1 | LPHN2* have been duplicated by an old SSD, while *EMR1, EMR2* and *LPHN1, EMR3* have been duplicated by recent SSDs. It implies that the entire region having *ELTD1 | LPHN2* genes was duplicated by the genome duplications. Duplication time are taken from Ensembl Compara. A link to the corresponding ohnolog family in other vertebrates has also been provided for each gene request, along with the association with human diseases from GeneCards [43] and COSMIC [44] databases.
(TIF)
