# Supplementary material for: Identification of Ohnolog Genes Originating from Whole Genome Duplication in Early Vertebrates, Based on Synteny Comparison across Multiple Genomes
Source: PLoS Comput Biol. 2015 Jul 16;11(7):e1004394. doi: 10.1371/journal.pcbi.1004394 (PMC4504502; doi:10.1371/journal.pcbi.1004394)
Supplement: S1 Text — (PDF) [file pcbi.1004394.s001.pdf]

# Supplementary Materials

## Identification of ohnolog genes originating from whole genome duplication in early vertebrates, based on synteny comparison across multiple genomes

Param Priya Singh[1], Jatin Arora[1], Hervé Isambert[1]

1 CNRS UMR168, UPMC, Institut Curie, Research Center, 26 rue d'Ulm, 75005, Paris France

## Supplementary Methods

### Identification of ohnologs

We used a gene-content based approach to detect the 2R-WGD retained sister regions having ohnologs, called synteny blocks, between a pair of outgroup (invertebrate) and paleopolyploid (vertebrate) genomes (Figure 1). Each vertebrate genome was compared to six outgroup genomes (outgroup comparison), to itself (self-comparison) and to the other vertebrate genomes. A flowchart summarizing our algorithmic approach is depicted in Figure 2.

#### Input genomes (Figure 2A)

We identified ohnologs from early vertebrate 2R-WGD in six amniote genomes, namely, human (*Homo sapiens*), chicken (*Gallus gallus*), dog (*Canis lupus familiaris*), pig (*Sus scrofa*), mouse (*Mus musculus*) and rat (*Rattus norvegicus*), by integrating multiple comparisons of content-based synteny conservation of each vertebrate genome relative to six invertebrate outgroup genomes, namely, one lancelet (cephalochordate): Amphioxus (*Branchiostoma floridae*), two tunicates (urochordates): *Ciona intestinalis* and *Ciona savignyi*, an echinoderm: sea urchin (*Strongylocentrus purpuratus*), and two basal bilaterians: fly (*Drosophila melanogaster*) and worm (*Caenorhabditis elegans*), Figure S4.

#### Protein coding genes and their genomic coordinates

We limited the analysis to protein coding genes. Except for sea urchin and Amphioxus, protein coding genes and their genome positions were obtained from Ensembl version 70 [33] using BioMart. Sea urchin and Amphioxus genes and their genome coordinates were downloaded from Ensembl Metazoa [34] and DOE Joint Genome Institute (JGI) [17] respectively. We further excluded genes belonging to unassembled scaffolds or haplotype regions in the vertebrate genomes. Each outgroup and vertebrate genome was then represented by a list of gene identifiers sorted on the basis of their start positions on their respective chromosome.

#### Orthologs and paralogs and duplication timing

Using unidirectional pairwise BLAST$p$ ($E$-$value < 10^{-5}$), we selected, for each vertebrate gene, its best orthologous match in a given outgroup genome. We then identified vertebrate genes sharing the same ortholog in this outgroup genome and considered them

as the only gene duplicates in the vertebrate genomes, for this specific outgroup-vertebrate synteny comparion.

The duplication timing of these paralogous genes within the vertebrate genomes were obtained from Ensembl compara [35] using BioMart. We noticed, however, ambiguities in duplication times and associated nodes in successive Ensembl versions. This is because Ensembl compara assesses duplication times by constructing gene families through clustering, and then reconciles gene trees for each family with the species tree. Therefore, as new organisms are added in updated versions, duplication nodes of paralog pairs can change. For example, the duplicate pair *RalGDS* – *RGL4* has been annotated to Eutheria in Ensembl v66, Bilateria in v67, again to Eutheria in v68-69 and to Euteleostomi in v70.

Therefore, rather than using just one node at the base of vertebrates, we considered paralogs from four duplication nodes at the base of vertebrates (Chordata, Vertebrata, Euteleostomi and Sarcopterygii), as candidate ohnologs with acceptable duplication time, before filtering them further based on synteny criteria (see below). For the human genome, we further took the consensus of 6 Ensembl releases (v65-v70) and collected paralog pairs whose duplication times were annotated to one of these four nodes in the majority of these Ensembl releases, taking the version v70 in the case of ambiguities. Figure S3 lists the number of genes, orthologs and paralogs for all the analyzed genomes.

### Identification of synteny blocks and anchors (Figure 2B)

A content based synteny block is defined as a region between an outgroup and a vertebrate genome (Figure S5A), or between two regions within the same vertebrate genome (Figure S5B) having multiple homologous gene pairs. Between the genomes of two species such blocks represent conserved genomic regions descended from their last common ancestor. Within the genome of the same organism, synteny blocks represent duplicated sister regions, provided the duplication time of the genes residing on such blocks is the same.

Vertebrate WGDs are among the oldest known genome duplications and the conservation of gene order or collinearity is limited [17]. However, conservation of macro- or content-based synteny can be observed between genomic regions, where there is a statistical enrichment of orthologs, even after more than 500 million years of independent evolution since the divergence between vertebrates and invertebrates [17, 31].

We used a window based approach to detect such regions between outgroup and vertebrate genomes extending earlier similar approaches [3, 32]. Any two regions between an outgroup and a vertebrate genome were considered to be candidate synteny regions (necessary but not sufficient condition), if there were at least $m$ orthologous gene pairs between them, within a window of size $W$, where $2 \leqslant m \leqslant W$. We scanned the genomes of invertebrate and vertebrate organisms by placing a symmetric window around the ortholog genes in each genome in such a way that there are $W/2$ genes upstream and downstream (Figure S5A). Hence, the ortholog partner under consideration is at the center of the windows in each genome. All such blocks were identified genome-wide and were labelled by the ortholog pair at the center of the blocks, referred to as the anchors, *e.g.* $O_7 - V_7, O_7 - V_7'$ in Figure S5A. At the chromosome boundaries, we kept the window size fixed by making it asymmetric around the anchor gene to avoid biasing the calculation of synteny *P-values* described below.

The procedure was repeated between regions in the same genome to perform the self comparison of vertebrate genomes and to identify all *vertebrate-vertebrate* anchors, *e.g.* $V_7 - V_7'$ in Figure S5B. While comparing two regions within the same vertebrate genome, we only considered paralogs duplicated at the base of vertebrates for each of the vertebrate genome according to Ensembl compara as detailed above.

### Calculation of P-value to rule out spurious synteny (Figure 2C)

Since we resort to content based synteny for different value of $W$ and $m$, it is important to establish that the observed synteny is not just by chance especially for large $W$ and small $m$. We detail below the estimation of the *P-values* for the observed content-based synteny between a vertebrate and a invertebrate windows of the same size $W$. The same approach can be readily applied to assess content-based synteny by self-comparison within the same vertebrate genome.

Given the ortholog pairs and their locations on the outgroup and vertebrate genomes, we calculate the probability of finding at least $k$ other orthologous gene pairs by chance between the same windows ($P_{\geqslant k} \equiv P - value$) for all identified anchors, as follows.

For any gene $O_i$ in the outgroup window (*e.g.* $O_i = O_5 : O_9$; Figure S6), we first calculate the probability $P_i$ of finding at least one ortholog of gene $O_i$ by chance in a random window of the same size $W + 1$ in the vertebrate genome, as,

$$P_i = 1 - \frac{\sum_s (l_s - W)}{\sum_c (N_c - W)}$$

where, $N_c$ is the total number of genes on the vertebrate chromosome $c$ and, $l_s$, the length of the segments $s$ of consecutive genes in the vertebrate genome without any ortholog of the outgroup gene $O_i$. Hence, $\sum_s (l_s - W)$ is the number of windows of size $W + 1$ in the vertebrate genome without any ortholog of the outgroup gene $O_i$ (*i.e.* blue segments of length $W + 1$ on the vertebrate genome between ortholog of $O_8$ in Figure S6).

We calculate the probabilities $P_i$ for all the genes $O_i$ in the outgroup window having ortholog(s) anywhere in the vertebrate genome (Figure S6). These probabilities are then used to estimate the *P-values* ($P_{\geqslant k}$) of finding at least $k$ other orthologous gene pairs by chance between the same windows.

This is illustrated on the example of Figure S6 with three additional ortholog pairs (green) between the two windows (boxes) in addition to the central anchor pair (red). Therefore, the *P-value* for the observed synteny is the probability that we find by chance 3 or more other orthologous genes in the same window of the vertebrate genome. In principle, we can directly calculate the probability of finding *any* combinations of 3, 4, 5,... genes in a given window by chance. However, for realistic window sizes (100 to 500), this becomes computationally unfeasible, as the number of combinations increases super-exponentially with the number of orthologs. Therefore, we resort to a faster mean field computation, assuming that the probabilities $P_i$ in the block are comparable and can be averaged over all outgroup genes with orthologs in the vertebrate genome (*i.e.* $P_{O5}, P_{O8}, P_{O9}$ in the example of Figure S6) excluding the anchor (*i.e.* $P_{O7}$ in Figure S6), as,

$$\log(\overline{P}) = \frac{1}{N_0} \sum_{i \neq anchor}^{N_0} \log(P_i)$$

where $\overline{P}$ is the geometric average of $P_i$ (excluding the anchor) for the window under consideration. The probability of observing $\geqslant k$ genes where $k$ is the observed orthologs between the two windows can then be estimated using the following binomial formula.

$$\begin{aligned} P_{\geqslant k} &= \sum_{j=k}^{N_0} \left[ \begin{pmatrix} N_0 \\ j \end{pmatrix} \times \overline{P}^j \times (1 - \overline{P})^{N_o - j} \right] \\ &= 1 - \sum_{j=0}^{k-1} \left[ \begin{pmatrix} N_0 \\ j \end{pmatrix} \times \overline{P}^j \times (1 - \overline{P})^{N_o - j} \right] \end{aligned}$$

where, $N_o$ is the number of outgroup genes in the outgroup window having at least one ortholog somewhere in the vertebrate genome (excluding the anchor pair).

This content-based synteny probability, $P_{\geqslant k}$, can be interpreted as the *P-value*, $P_{O-V}$, assessing our confidence on the synteny of the outgroup and vertebrate synteny regions around the anchor pair, $O - V$. The same approach can be readily adapted to compare two windows within the same vertebrate genome for self-comparison synteny identification (see below).

### Identify putative ohnolog pairs (Figure 2D)

Due to the two rounds of genome duplication in the vertebrate genome, each synteny window in the outgroup genome should ideally correspond to up to four windows in the vertebrate genome, however, only a minority of ohnologs are in fact retained in more than 2 copies (see final counts in Table 1 & Figure S2 for numbers).

To search for ohnolog pair candidates, we identify anchors in the vertebrate genome that share the same outgroup gene (*e.g.* $O_7 - V_7$ and $O_7 - V_7'$ in Figure S5A). Vertebrate genes with such overlapping anchors define the initial ohnolog pair candidates. Yet, the duplication time of these ohnolog candidates may not correspond to the base of vertebrates (in particular for very relaxed synteny criteria). Such initial ohnolog candidates, that are not duplicated at the correct time according to Ensembl, are excluded from our list of ohnolog pair candidates. The remaining ohnolog pair candidates are then further filtered by combining their *P-values* relative to the different outgroups, as detailed below.

By contrast, for vertebrate genome self-comparison, since we have restricted the analysis to paralogs from the base of vertebrates, each anchor pair can be directly taken as ohnolog pair candidate (*e.g.* $V_7 - V_7'$ in Figure S5B), before its statistical significance can be assessed as described below.

### Define q-scores by combining P-values from anchors (Figure 2E)

For outgroup comparison, candidate ohnologs correspond to two anchor pairs sharing the same orthologous gene in the outgroup genome. Thus, the statistical confidence of each candidate ohnolog pairs must *a priori* consider two *P-values* corresponding to each anchor. For example, for the ohnolog candidate pair $V_7 - V_7'$ in Figure S5A, we obtain two *P-values*, $P_{O_7-V_7}$ and $P_{O_7-V_7'}$, from each anchor. These two *P-values* can be very different as they depend on different genomic contexts in the vertebrate genome. In the following, we have taken the largest *P-value* from the least conserved synteny block as *quantitative* synteny assessment or *q-score*, $Q_{\,V-V'_{\rm og}}$, for each ohnolog pair candidate,

$$Q_{\,V-V'_{\rm og}} = \max(P_{O-V},\ P_{O-V'})$$

Note that this *q-score* definition is more stringent than a standard *P-value* by biasing the quantitative synteny assessment of ohnolog pair candidates towards the largest *P-value* of the two synteny comparisons. However, the smallest *P-value* corresponding to the most conserved synteny block may solely reflect shared ancestry without WGD between the two lineages.

Similarly, for self comparison, because synteny comparison is directional, we also get two *P-values* (*e.g.* for $V_7 \to V_7'$ and $V_7' \to V_7$ comparisons in Figure S5B), each block being alternatively chosen to play the role of the outgroup region to calculate a *P-value* as above. We take the geometric mean of these two *P-values* as *quantitative* synteny assessment for self comparison, $Q_{\,V-V'_{\rm self}}$, for each ohnolog pair candidate (*e.g.* for $V_7 - V_7'$ in Figure S5B),

$$\log(Q_{\,V-V'_{\rm self}}) = \frac{1}{2}\big(\log(P_{V\to V'}) + \log(P_{V'\to V})\big)$$

### Sample genomes with multiple window sizes (Figure 2F,G)

Typically, window based approaches for inferring synteny only consider a single window size [3, 32]. However, there is *a priori* no optimum size of the window. Since we have a quantitative assessment of the statistical significance of content-based synteny, we have repeated and integrated the above procedure using multiple window sizes (*i.e.* steps A-E in Figure 2). We start with a relatively small window size of 100 and sample each genome with increasing window sizes of 200, 300, 400 and 500, with a minimum of $k = 2$ orthologs/paralogs required between each windows (including the anchor).

If an ohnolog pairs is identified by multiple window sizes, $w$, we obtain an effective q-score, $Q_{\overline{w}}$, for that pair using the geometric mean of the q-scores, $Q_w$, from all the window sizes by which the pair is identified,

$$\log(Q_{\overline{w}}) = \frac{1}{n_w} \sum_{w}^{n_w} \log(Q_w)$$

where $n_w$ is the number of window sizes by which the pair can be identified. We compute the average q-scores and store all the ohnolog pairs for each outgroup ($Q_{\overline{w}_{\mathrm{og}}}$) and self ($Q_{\overline{w}_{\mathrm{self}}}$) comparison.

For self comparison, these average q-scores ($P_{\overline{w}_{\mathrm{self}}}$) can then be directly used to assess statistical confidence of the content based synteny,

$$Q_{\mathrm{self}} \equiv Q_{\overline{w}_{\mathrm{self}}}$$

whereas the average q-scores for outgroup comparison ($P_{\overline{w}_{\mathrm{og}}}$) will be further improved by integrating multiple outgroup comparisons to enhance the statistical confidence of the content-based synteny, as detailed below.

### Combine q-scores from all outgroups (Figure 2H)

We perform comparison of each vertebrate genome with six different outgroups to overcome lineage specific rearrangements in the different outgroups. In addition, if an ohnolog pair is identified by multiple outgroups, it strengthens our confidence that the pair is a 'true' ohnolog. Therefore, we assess the likelihood of ohnolog pairs from multiple outgroups by multiplying their q-scores from all outgroups,

$$Q_{\mathrm{outgr}} \equiv \prod_{\mathrm{og}}^{all\ outgroups} Q_{\overline{w}_{\mathrm{og}}}$$

This amounts to assume that the synteny conservation is independent for different outgroups due to lineage specific rearrangements since their divergence more than 500 MY ago. In fact, comparisons with randomized genomes, Figure S7, confirmed limited spurious identification of false positive ohnologs due to outgroup genome correlations. All in all, using multiple outgroups improves the statistical significance of the inferred ohnolog pairs in each vertebrate.

### Average q-scores over amniote genomes (Figure 2I)

In addition to rearrangements in outgroup genomes, lineage specific rearrangements in vertebrate genomes can also hinder the identification of ohnologs. To circumvent this difficulty, we took advantage of ohnolog pairs identified in multiple amniote genomes. We then took the geometric average of their q-scores over amniotes sharing these ortholog pairs in Ensembl.

In case of multiple such ohnolog pairs due to lineage specific SSD, we used the best ohnolog pair having either minimum q-score from outgroup synteny, or self synteny,

or having maximum outgroup support in the genome with multiple pairs. We then calculated the geometric mean of q-scores for self and outgroup synteny comparisons and assigned them to each consensus ohnolog pair.

This yields the final averaged q-scores for outgroup ($\overline{Q}_{\text{outgr}}$) and self-synteny ($\overline{Q}_{\text{self}}$) comparisons, for all ohnolog candidate pairs identified in at least one amniote genomes. Using averaged q-scores improves the statistical significance of the inferred ohnolog pairs by circumventing some recent lineage specific rearrangements in amniote genomes, while taking into account their long common evolutionary history since divergence from invertebrate outgroups. The integration allows us to identify genes that are no longer in significant synteny in a particular vertebrate genome, but are ohnologs with very high confidence in other vertebrates.

### Filter ohnolog pairs to remove false positives (Figure 2J)

For each pair that is identified by both the outgroup and self comparisons, we obtain two q-scores. Any custom criteria can now be used to filter high confidence ohnologs. Three different synteny criteria, combining q-scores from both outgroup comparison and self-comparison, have been used throughout this study,

- **Strict**: $\overline{Q}_{\text{outgr}} < 0.01$ AND $\overline{Q}_{\text{self}} < 0.01$
- **Intermediate**: $\overline{Q}_{\text{outgr}} < 0.05$ AND $\overline{Q}_{\text{self}} < 0.3$
- **Relaxed**: $\overline{Q}_{\text{outgr}} < 0.05$ OR ($\overline{Q}_{\text{outgr}} < 0.5$ AND $\overline{Q}_{\text{self}} < 0.01$)

These three statistical criteria represent decreasing confidence in the ohnolog status of the identified ohnolog pairs. In principle, the relaxed criteria may also include a number of paralogs from large scale segmental duplicates from the origin of vertebrates, as paralog pair candidates have been filtered with duplication time at the base of vertebrates (see above).

### Construction of ohnolog families (Figure 2K)

Ohnolog families are built from the filtered ohnolog pairs from strict, intermediate and relaxed criteria, above. Due to the two rounds of WGDs, we expect that most of these ohnolog families should be of size 2, 3 and 4. However, SSDs and large scale segmental duplicates may lead to family sizes larger than four. All paralogs from Ensembl compara which could not be identified as ohnologs were assumed to be SSD duplicates. These SSD duplicates correspond to duplicates from all ages, including before and after the 2R-WGD.

To construct ohnolog families, we start with an ohnolog pair and use a depth first search algorithm (DFS) [36]. DFS is an algorithm used for traversing a tree or graph structure. Starting from ohnolog pairs, we identified separate sets of connected ohnolog pairs corresponding to different ohnolog families. To construct such ohnolog families for all the pairs using DFS, we started with any ohnolog pair as the root node and recursively explore all branches until no new ohnologs can be found. We marked the ohnologs already visited to avoid traveling along the same path again and backtracked to follow new ohnolog branches iteratively until no additional ohnologs can be identified in each ohnolog family. However, ohnolog families constructed using this exhaustive approach may contain gene pairs, $G_1$ and $G_2$, which are SSD with respect to each other but are ohnolog partners of a third gene, $G_3$, in the family. We identify and display such SSDs with different separator symbols depending on their duplication time relative to the 2R-WGD.

- *i)* $G_1|G_2$: correspond to old SSD paralogs, duplicated before or around the same time of the 2R-WGD and lying less than 100 genes (smallest window in our analysis) apart on the same chromosome.
- *ii)* $G_1, G_2$: correspond to more recent SSD paralogs, duplicated after the 2R-WGD and lying less than 100 genes apart on the same chromosome.

Therefore, the final ohnolog families consist of ohnolog partners along with the information on recent and/or old SSD if the ohnolog has undergone additional duplication episodes according to Ensembl family trees, see *e.g.* the human *EMR3* family in Figure S13.

### Comparison with randomized human genome

To verify that our algorithm inferring ohnolog pairs, based on the multiplication of outgroup q-scores, incorporates only a limited number of false positive ohnolog pairs, we performed the same analysis after randomizing the gene order in the human genome. To this end, we shuffled the human genome to place each gene on a random chromosome at a random position. Keeping chromosome size, ortholog and paralog relations fixed, we then repeated our approach to identify ohnolog pairs and calculate their combined q-scores for windows of size 300 genes in the randomized human genome and the original outgroup genomes.

We then compared the q-scores obtained with the original and shuffled human genomes. The q-score distributions (25 bins) for both scenarios are depicted in (Figure S7).

In Figure S7A, we have first restricted the outgroup q-score estimates to the four least distant outgroup genomes by excluding the two basal bilaterians (fly and worm). We observed that there is hardly any enrichment of q-scores in the lower probability bins after random shuffling of the human genome, by contrast with the sharp increase observed with the actual human genome (corresponding to the inferred ohnolog pairs in the actual human genome).

In Figure S7B, all six outgroups have been included for the estimation of outgroup q-scores and we observe a limited enrichment of gene pairs in the lowest q-score bins after human genome shuffling, compared with the original human genome. This small enrichment in the lowest q-score bins is related to the abundance of lineage specific SSD duplicates that occurred in fly and worm (this leads to a large initial number of human orthologs in these genomes, Figure S1).

Hence, all in all, these comparisons between shuffled and original human genomes demonstrate that our q-score estimates, combining content based synteny from several outgroups, improve the statistical identifications of ohnologs, while incorporating only a limited number of false positive ohnologs in the resulting ohnolog dataset.

### Collection of cancer, disease and autoinhibitory genes

The genes mutated in cancers were obtained from multiple databases including COSMIC (v64) [40], as described in [5]. We divided all cancer genes into two subsets: core and putative cancer genes. Core cancer genes either had experimental evidence of mutations in cancers or were highly mutated in the COSMIC database ($\geqslant 5$ non-synonymous mutations). Putative cancer genes are either from COSMIC (non-synonymous mutations $\geqslant 1$ and $\leqslant 5$) or from text search in multiple databases [5]. Specific mutations in cancer genes from the core dataset are either known or strongly suspected to drive cancer progression, while the causal link is more uncertain for genes in the putative dataset. In total, we obtained 8,899 cancer genes with 2,743 Core and 6,156 Putative cancer genes.

We obtained 5,172 human disease genes from Gene Cards [43] database. Using the inheritance patterns from OMIM, we could obtain 679 and 888 genes that were unambiguously described as autosomal dominant and autosomal recessive genes respectively.

A first set of 461 genes with autoinhibitory protein folds were obtained as described in [5]. Namely, we performed literature search in PubMed with keyword "autoinhibitory domain" and retrieved relevant genes through a careful manual curation of the articles. We also searched "auto/self-inhibit*" in various databases (OMIM, SwissProt, NCBI Gene and GeneCards), and identified additional candidates after manual curation of the list of genes [5]. Finally, we ran a Hidden Markov Model (HMM) search against Pfam database [42] to identify domains in all the human genes using HMMER [41], and included the genes having known domains frequently implicated in autoinhibition (*i.e.* SH3, DH, PH, CH, Drf and Eth domains). This led to a total of 881 genes with autoinhibitory protein folds.

### Gene Ontology (GO) enrichment analysis

GO enrichment analysis was performed using DAVID [39], with the ohnologs from the relaxed criteria for each organism. For dog and pig ohnologs, no significant enrichment was obtained due to lack of proper GO annotations for these organisms. Bonferroni multiple correction was used to analyze enrichment.
