# Supplementary figures and images for: Identification of Ohnolog Genes Originating from Whole Genome Duplication in Early Vertebrates, Based on Synteny Comparison across Multiple Genomes
Source: PLoS Comput Biol. 2015 Jul 16;11(7):e1004394. doi: 10.1371/journal.pcbi.1004394 (PMC4504502; doi:10.1371/journal.pcbi.1004394)

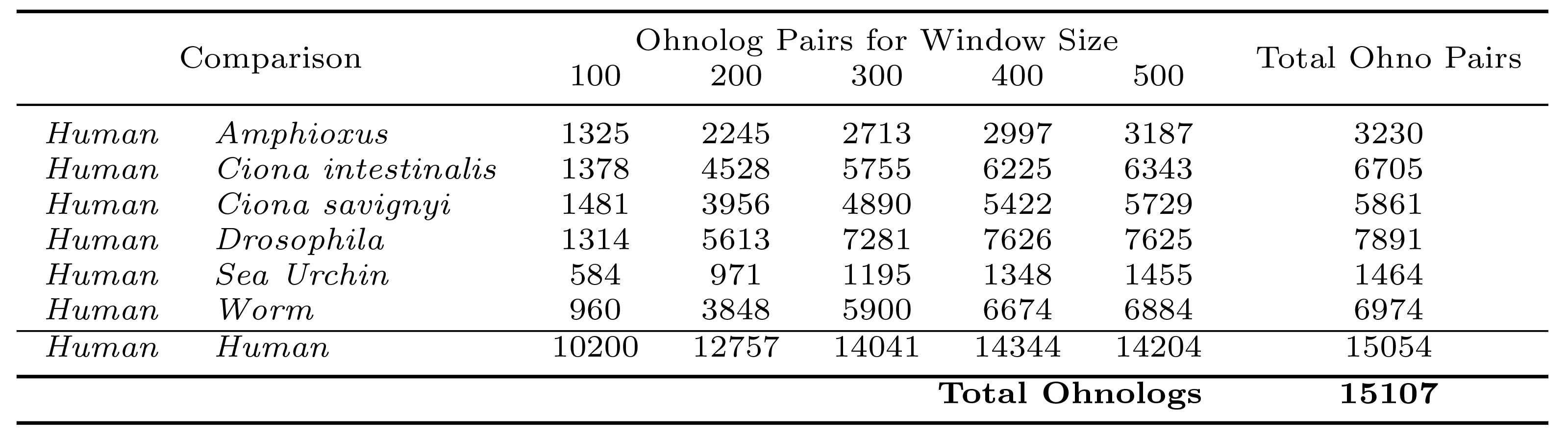

Supplement: S1 Fig — Number of human ohnologs identified by outgroup and self comparison before applying any quantitative filter for content-based synteny. (TIF) [file pcbi.1004394.s002.tif]

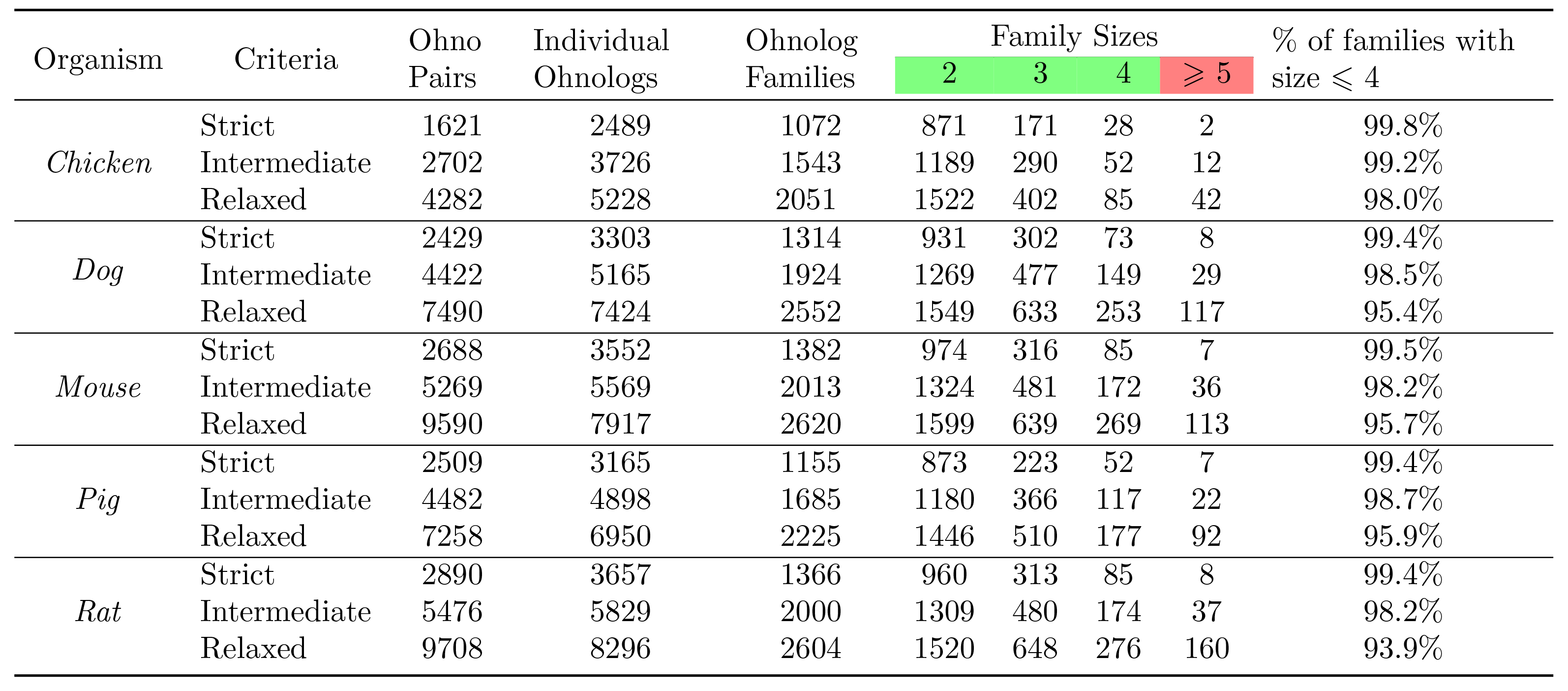

Supplement: S2 Fig — Individual ohnologs, pairs and families for the three quantitative criteria in the five non-human amniote genomes analyzed. (TIF) [file pcbi.1004394.s003.tif]

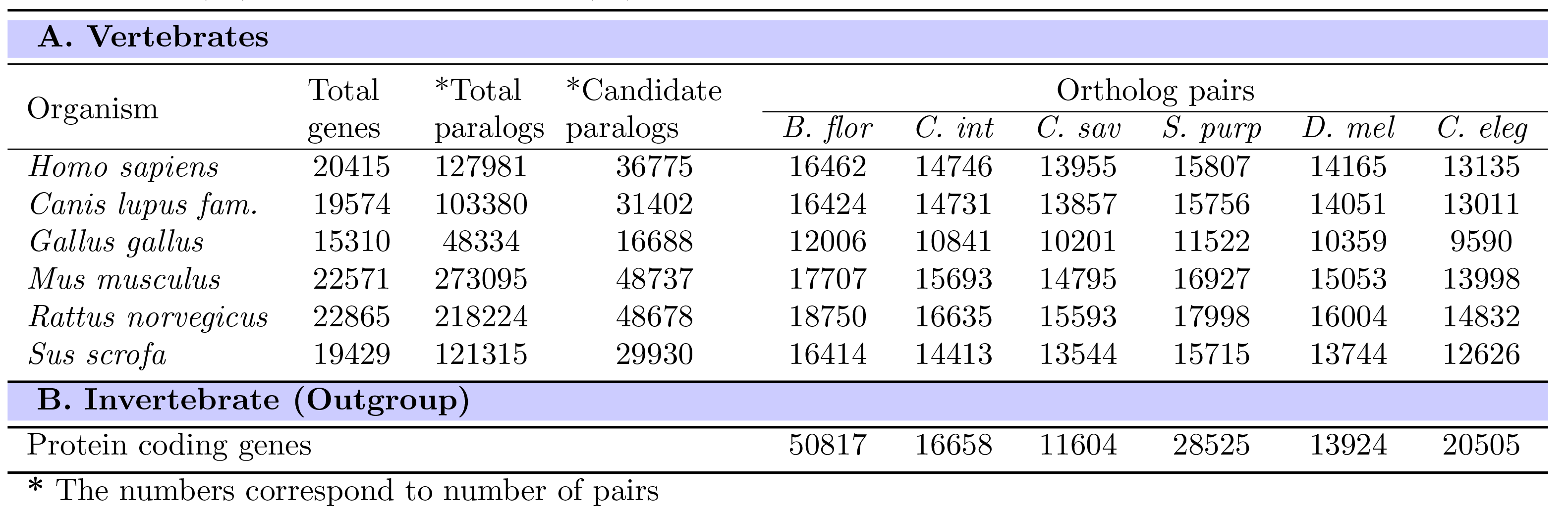

Supplement: S3 Fig — Number of protein coding genes, orthologs and paralogs for the analyzed vertebrate (A) and invertebrate (B) genomes. (TIF) [file pcbi.1004394.s004.tif]

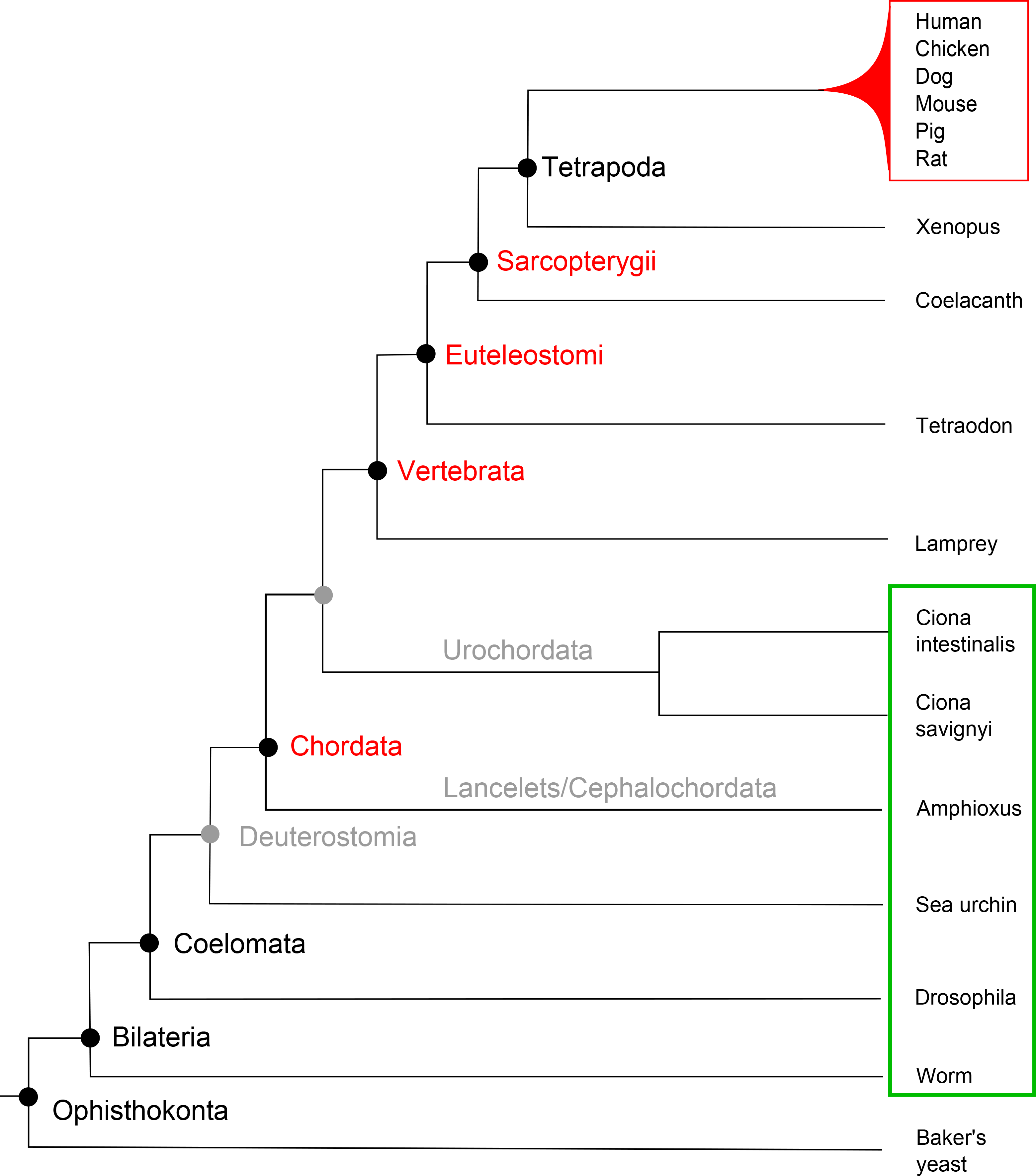

Supplement: S4 Fig — Schematic tree for the paleopolyploid and outgroup organisms with duplication nodes taken from Ensembl Compara [33–35]. Gray nodes are not part of Ensembl. Paleopolyploid vertebrate genomes included in this study are highlighted with a red box and invertebrate outgroups (for the 2R-WGD) are highlighted by a green box. (TIF) [file pcbi.1004394.s005.tif]

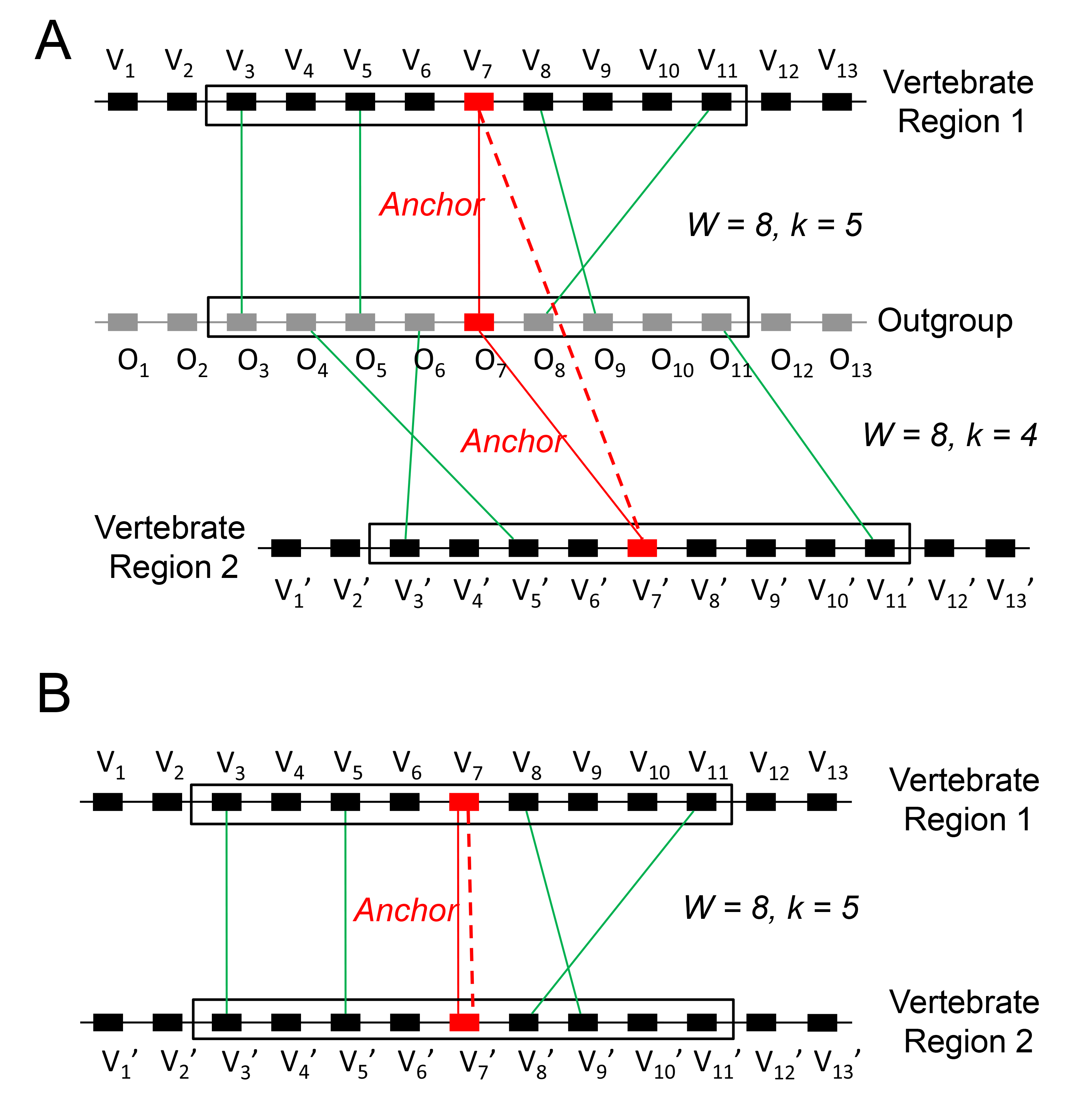

Supplement: S5 Fig — Comparison of genomic regions to identify anchor pairs (in red) and ohnolog candidate pairs (dashed red). Each block represents a gene labeled by O i on the outgroup genome and V i on the vertebrate genome. Duplicated regions in the vertebrate genome are marked by V1′−Vn′. Other orthologous (A) and paralogous (B) relations are depicted by green lines. (A) Identification of synteny anchors between an outgroup window and two windows in the vertebrate genome. Using a window of size 8(+1) centered around the O 7−V 7 and O7−V7′ orthologous pairs, we observe 4 and 3 additional gene pairs between the outgroup and the vertebrate regions 1 and 2, respectively. Hence, O 7−V 7 and O7−V7′ are two anchors sharing the same outgroup ortholog O 7. Hence V7−V7′ are inferred to be an ohnolog pair candidate, which will be further filtered with quantitative statistical significance criteria or q-score, Qoutgr, see text. (B) Identification of ohnologs between two regions in the same vertebrate genome. The anchor V7−V7′ having four additional paralog pairs between the windows, it is directly taken as an ohnolog pair candidate, to be further filtered with quantitative statistical significance criteria or q-score, Qself, see text. (TIF) [file pcbi.1004394.s006.tif]

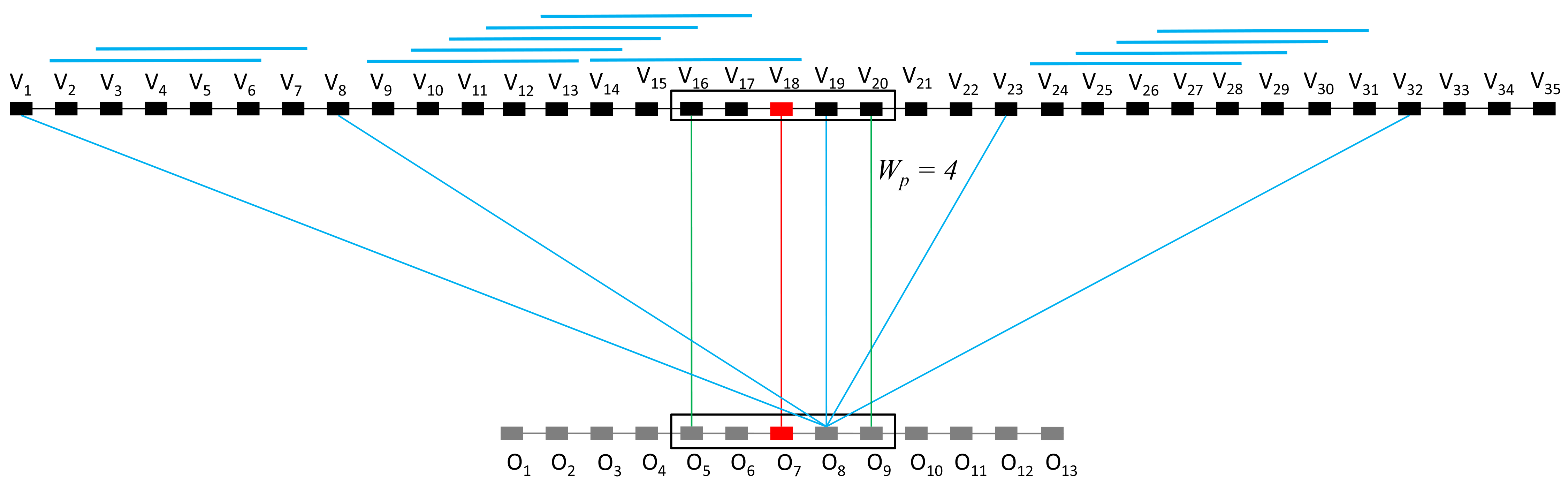

Supplement: S6 Fig — The calculation of P i for an outgroup gene O i. Illustration of the likelihood calculation, P i, for an outgroup gene O 8 to have an ortholog gene in the vertebrate window (V 16−V 20) defined by the anchor pair (O 7−V 18). O 8 has 5 orthologs in the vertebrate genome: V 1, V 8, V 19, V 23 and V 32. There are 12 possible window locations (highlighted in blue) without any of these orthologs in the vertebrate genome. P i for this anchor then becomes 1 − 12/31 = 0.6, where 31 is the total number of possible windows on this schematic vertebrate genome (N−W). (TIF) [file pcbi.1004394.s007.tif]

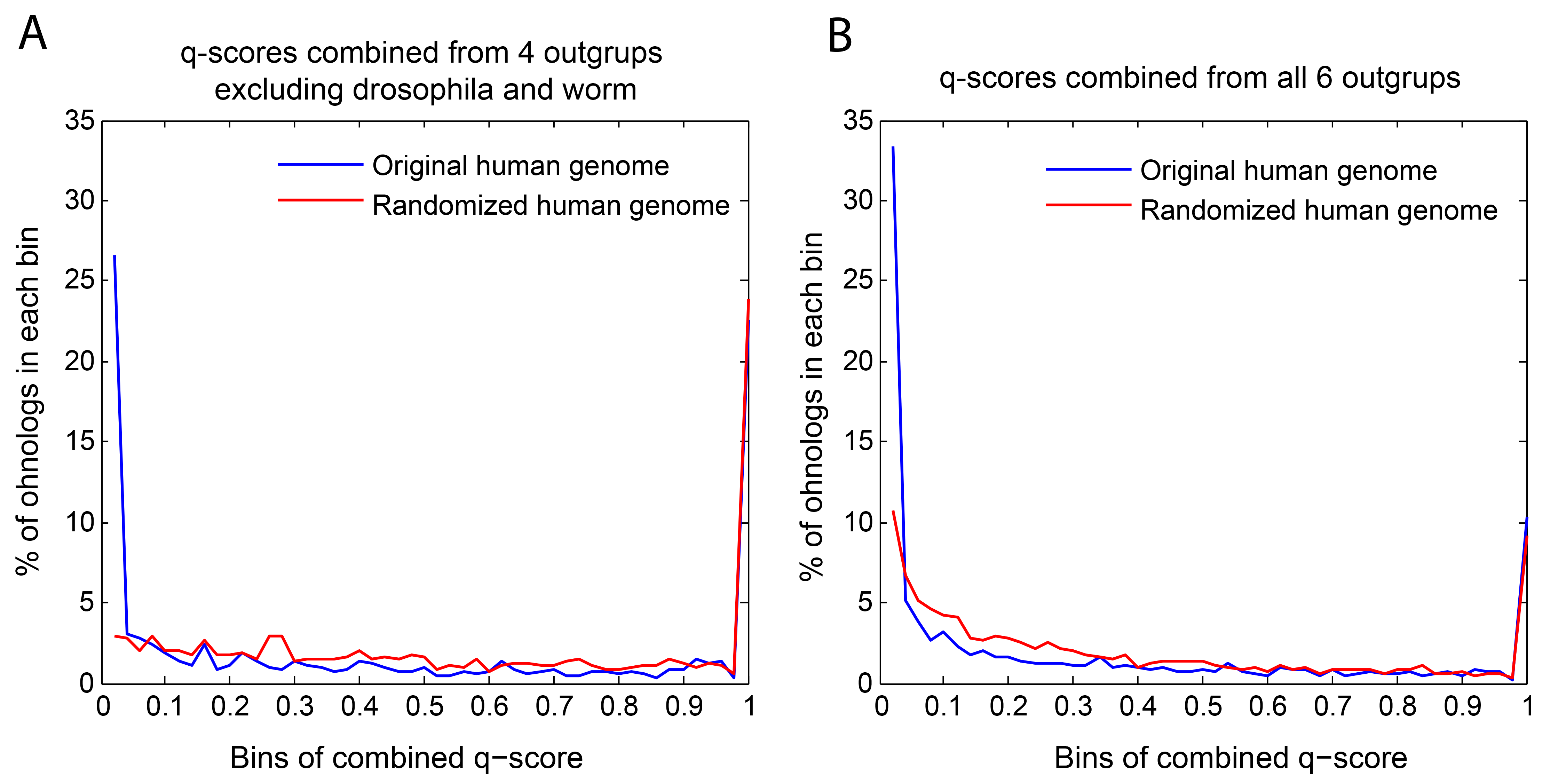

Supplement: S7 Fig — Comparisons of the global q-score distributions from the original (blue) and randomized (red) genomes; (A) without worm and fly outgroups; (B) with all six outgroup genomes. (TIF) [file pcbi.1004394.s008.tif]

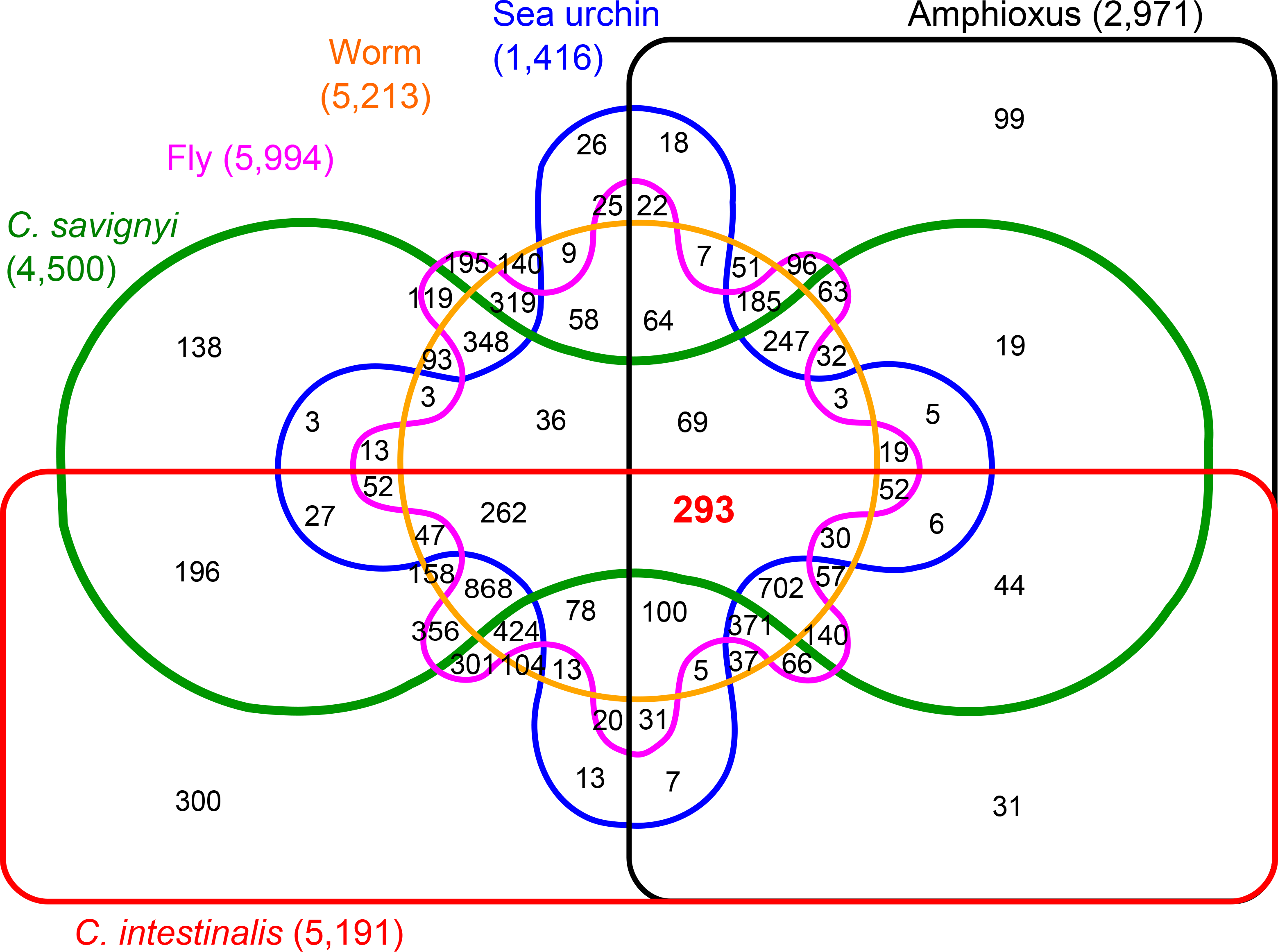

Supplement: S8 Fig — A six-way Venn diagram showing the distribution in numbers of the 7,715 human ohnolog pairs identified by at least one outgroup and predicted from the relaxed criteria. (TIF) [file pcbi.1004394.s009.tif]

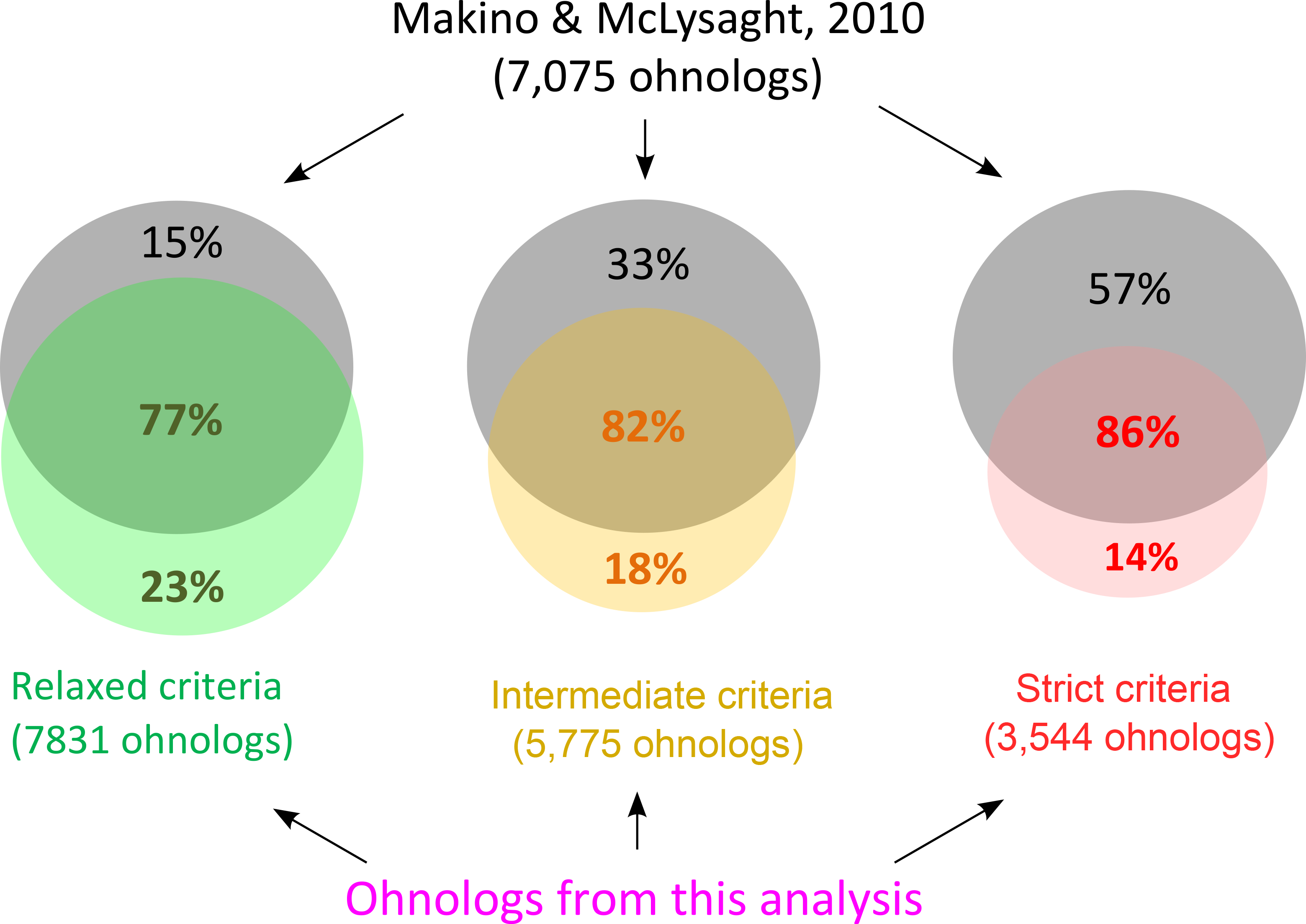

Supplement: S9 Fig — Comparison of our human ohnolog prediction for the three quantitative criteria (strict, intermediate and relaxed, see main text) and the ohnolog dataset from [3]. (TIF) [file pcbi.1004394.s010.tif]

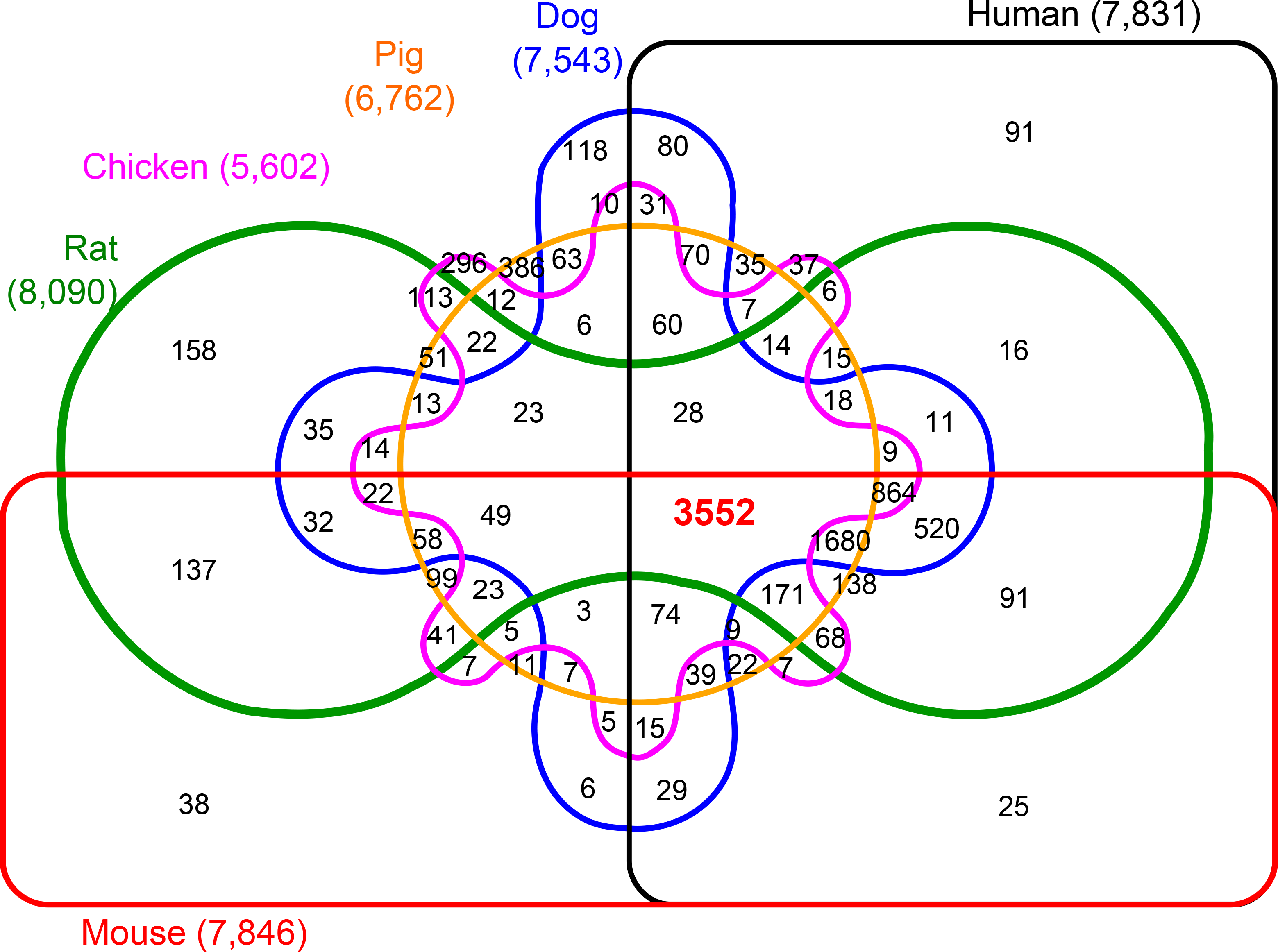

Supplement: S10 Fig — A six-way Venn diagram showing the distribution in numbers of the ohnologs identified in at least one amniote and predicted from the relaxed criteria. (TIF) [file pcbi.1004394.s011.tif]

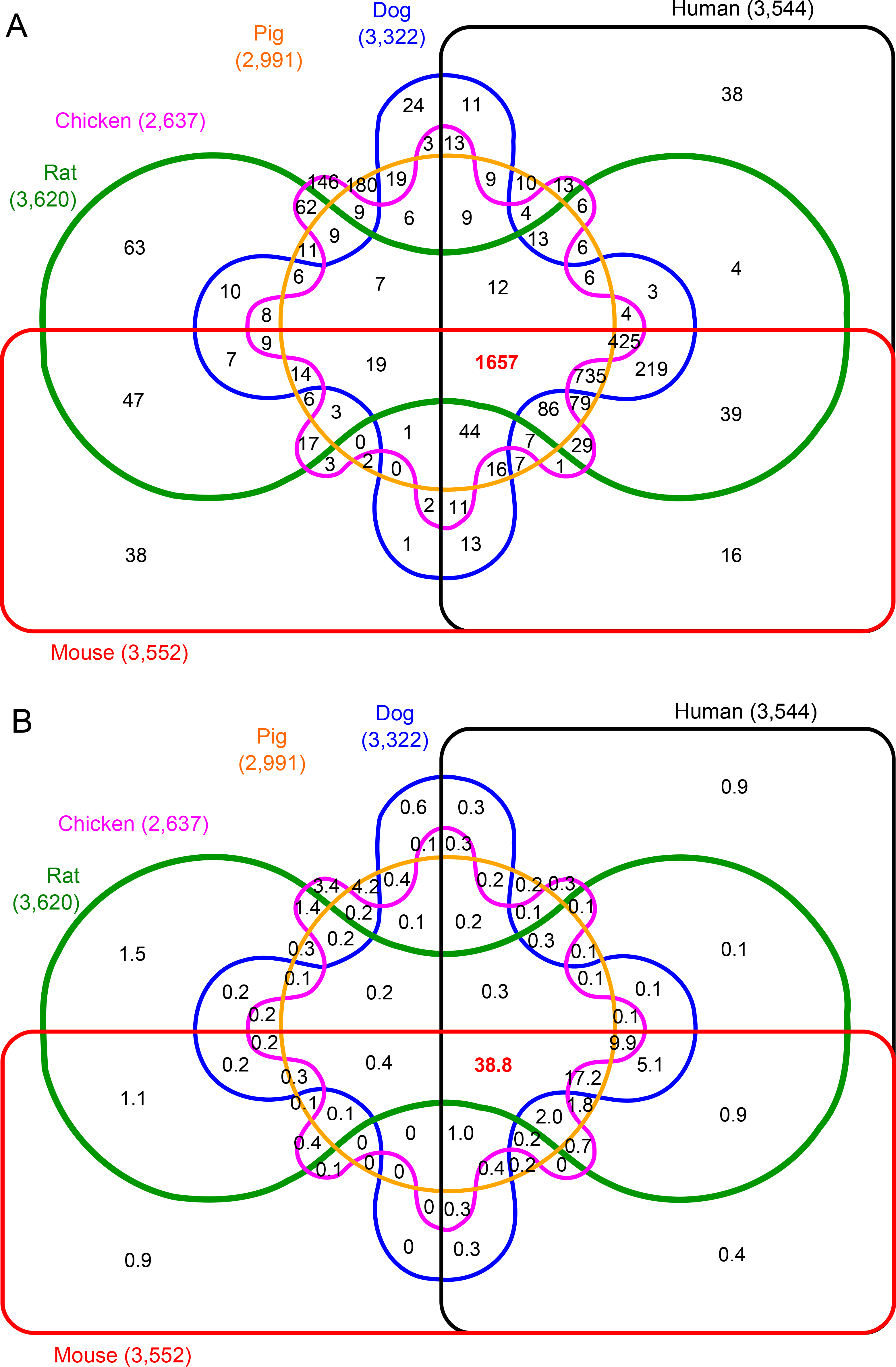

Supplement: S11 Fig — A six-way Venn diagram showing the distribution in numbers (A) and percentages (B) of the ohnologs identified in at least one amniote and predicted from the strict criteria. (TIF) [file pcbi.1004394.s012.tif]

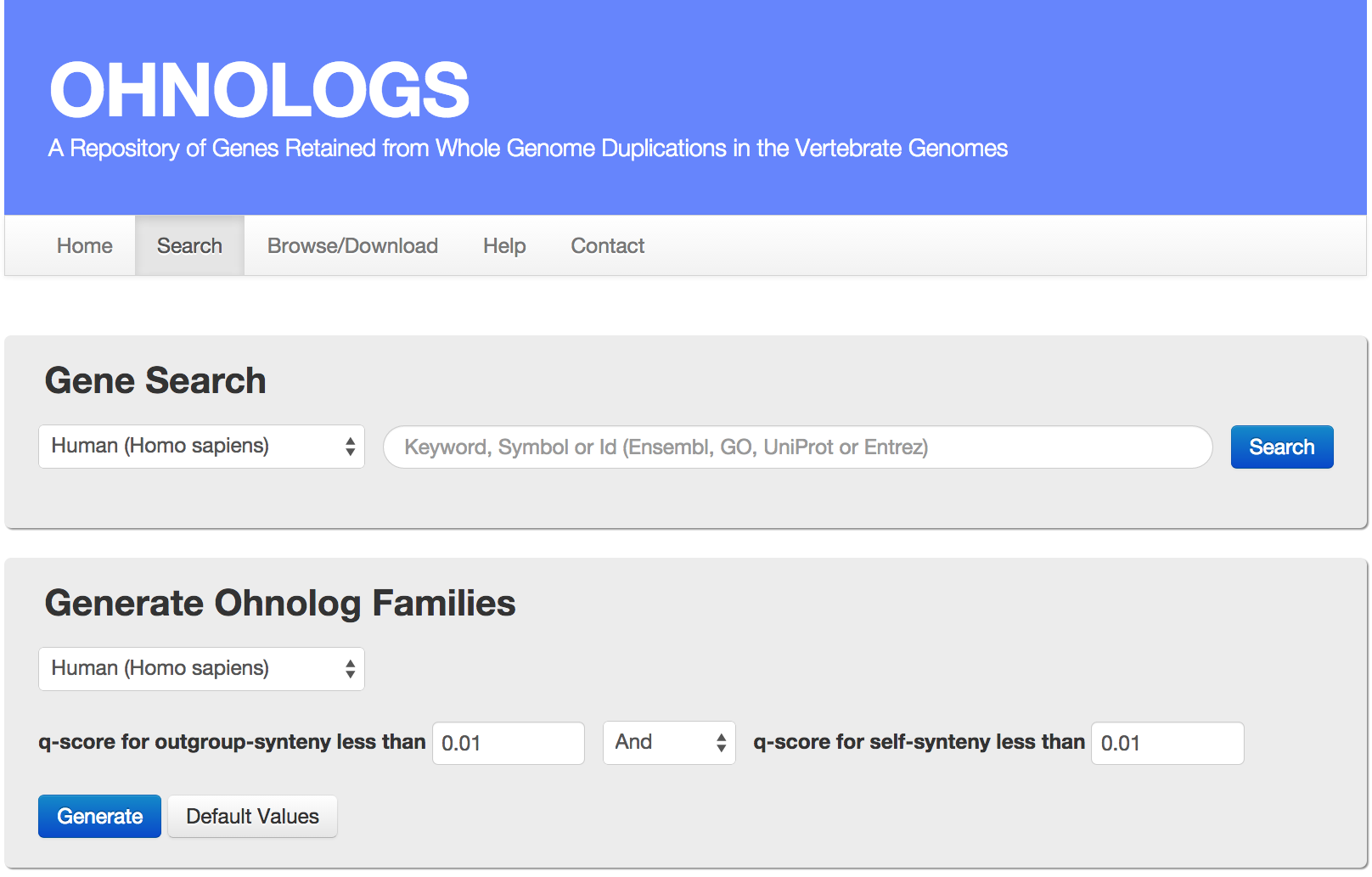

Supplement: S12 Fig — (TIF) [file pcbi.1004394.s013.tif]

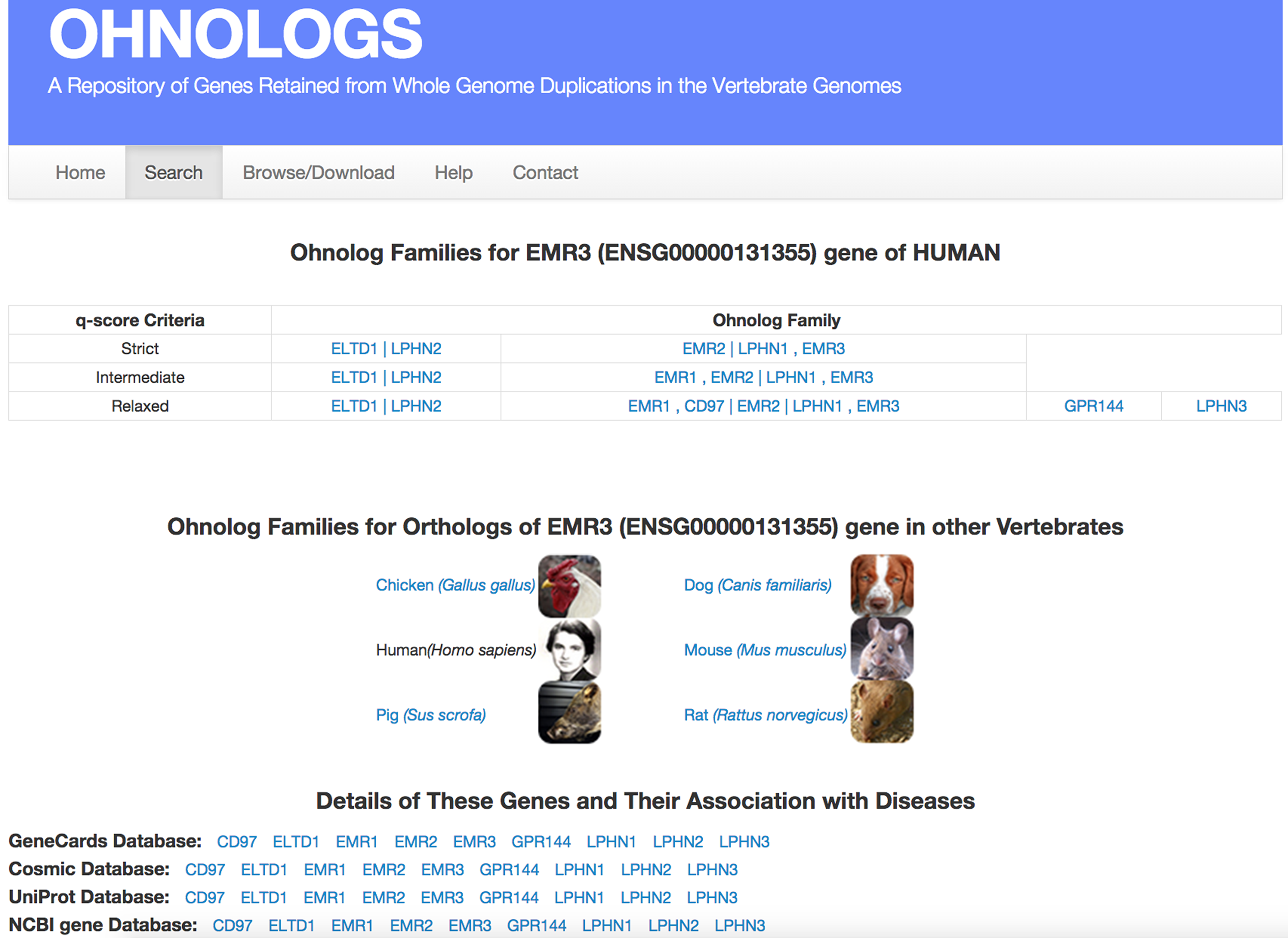

Supplement: S13 Fig — The result page of the ohnolog family search for the human EMR3 gene is depicted. Families from all three quantitative criteria are displayed, see text. Using the strict criterion, a family of size 2 is generated where ELTD1 & LPHN2 are ohnologs with EMR2, EMR3 & LPHN1. Relaxing the q-score to the intermediate criteria results in an additional ohnolog in this family, EMTR1; and to the relaxed criteria results in a family of size 4. Ohnolog partners for the families are displayed in different columns. Genes within the same cell are small scale duplicates e.g. ELTD1—LPHN2. We use two different separators for SSDs: a comma (,) to distinguish if it is a recent SSD (after 2R-WGD), and a pipe (|) for an ancient SSD (before or around the same time as the 2R-WGD). Hence, ELTD1 | LPHN2 have been duplicated by an old SSD, while EMR1, EMR2 and LPHN1, EMR3 have been duplicated by recent SSDs. It implies that the entire region having ELTD1 | LPHN2 genes was duplicated by the genome duplications. Duplication time are taken from Ensembl Compara. A link to the corresponding ohnolog family in other vertebrates has also been provided for each gene request, along with the association with human diseases from GeneCards [43] and COSMIC [44] databases. (TIF) [file pcbi.1004394.s014.tif]
